# A Review of Advancements in Metal Oxide Semiconductor Gas Sensors for Methane and Carbon Monoxide Towards Coal Mine Safety

**DOI:** 10.3390/ma19173808

**Published:** 2026-09-07

**Authors:** Qian Zhang, En-San Fu, Ze Yang, Le-Xiao Tian

**Affiliations:** 1 Information Institute of the Ministry of Emergency Management of the PRC, Beijing 100029, China; 2 Key Laboratory of Mine Major Disaster Risk Monitoring and Early Warning Technology National Mine Safety Administration, Beijing 100029, China

**Keywords:** metal oxide semiconductors, gas sensors, methane, carbon monoxide, coal mine safety

## Abstract

Underground coal mining operations remain significantly threatened by the accumulation of methane (CH_4_) and carbon monoxide (CO): Methane poses an acute explosion risk, and carbon monoxide serves as a critical biomarker for spontaneous coal combustion. Consequently, rigorous real-time monitoring to ensure environmental safety is necessitated, which is based on superior gas sensor devices. Although various detection modalities exist, conventional methods are frequently constrained by environmental sensitivity and limitations regarding long-term sensor stability. This review provides a comprehensive analysis of recent advancements in chemiresistive gas sensors based on metal oxide (MO) semiconductor materials with low cost, high stability, high sensitivity, and easy preparation, which are engineered for the detection of methane and carbon monoxide in coal mining environments. This study examines the redox-sensing mechanisms of both n-type and p-type MO semiconductors, for which special attention is directed toward optimization strategies designed to overcome the high activation energy of methane and improve carbon monoxide response kinetics. Importantly, novel approaches to lower high operating temperatures and improve the selectivity of MO sensors under complex mine environments have been comprehensively discussed.

## 1. Introduction

As one of the most hazardous industrial activities, coal mining is widely accompanied by environmental risks due to complex underground conditions, which can seriously threaten the lives of miners [1,2,3,4]. Among the numerous dangers present in underground coal mines, two gaseous hazards stand as dominant threats: methane (CH_4_) and carbon monoxide (CO) [5,6,7,8]. As a colorless and odorless hydrocarbon gas, methane is hardly discovered by miners during coal mining, during which methane is easily accumulated [9,10]. As a result, methane can pose an ever-present risk of explosion when its concentration reaches between 5% and 15% in air [11,12]. In year 2026, the methane explosion in Qinyuan county of China has caused the death of 82 people, with two missing persons, which is a harsh lesson for mine safety [13]. Identically dangerous, carbon monoxide is generated primarily through the oxidation of coal—a process that intensifies as temperatures rise during the early stages of coal spontaneous combustion, one of the most insidious fire hazards in mining environments [14,15,16,17]. The simultaneous presence of these gases creates a dual-threat scenario that demands constant vigilance and sophisticated monitoring solutions [18,19,20,21].

The imperative for real-time gas monitoring in coal mines is crucial for safety, as work cannot be conducted in environments where methane exceeds 2% or where carbon monoxide concentrations surpass 50 parts per million (0.005%) according to underground coal mine regulations [22,23,24,25]. It is critical to distinguish among the various regulatory thresholds, which serve different safety functions. In China, the alarm threshold for CH_4_ is typically set at 1% by volume, triggering a visual and audible warning. The action threshold for mandatory evacuation or equipment shutdown is generally specified at 1.5% CH_4_. The flammability limit of methane in air is approximately 5–15%, which defines the explosive hazard range and is used for broader risk assessments but is not a permissible operational concentration. For CO, a warning threshold of 24 ppm (0.0024%) is often used in Chinese coal mines to signal the onset of spontaneous combustion, while the action threshold for worker evacuation is 50 ppm (0.005%). These regulatory thresholds represent critical safety boundaries that signal different levels of imminent danger and demand distinct responses. Historically, the mining industry has relied on various gas detection methods to safeguard workers, but the limitations of traditional approaches have become increasingly apparent in the face of modern safety demands. Therefore, novel gas sensor devices with high sensitivity, high precision, and high stability are strongly demanded as fundamental components for real-time gas monitoring to guarantee mine safety [26,27,28].

As shown in Table 1, conventional gas detection techniques have included gas chromatography, electrochemical sensors, and catalytic combustion detectors [29]. While each method has contributed to mine safety, significant drawbacks widely exist [30]. Electrochemical sensors, for instance, are susceptible to interference from temperature and humidity fluctuations [31,32]—conditions that vary dramatically in underground environments. Catalytic combustion sensors, commonly used for methane detection, can be poisoned or inhibited by certain compounds, leading to sensor failure at critical moments [33]. These limitations have driven the search for more robust, reliable, and intelligent sensing devices and technologies [34].

**Figure 1:**
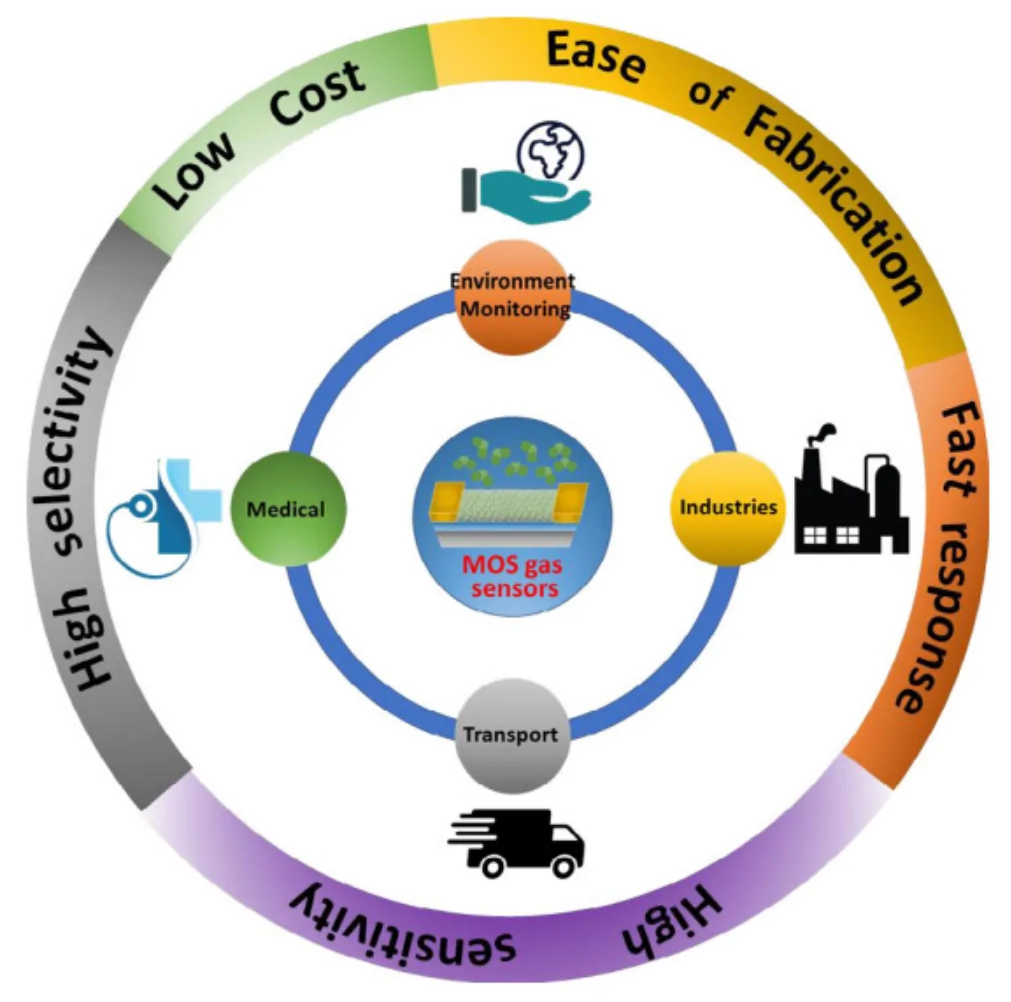
Application fields of MO-based gas sensors with inherent advantages. Reproduced from Reference [41], with permission from Wiley-VCH.

Sensors with metal oxide (MO) semiconductors have been widely used in various application scenes due to low cost, high stability, and long lifetime (Figure 1) [35]. The dominant sensing mechanism of MO semiconductors is the oxidation and reduction reactions when methane, a reducing gas, is absorbed onto the MO layer [36]. As a result, electrical conductivity can be modulated to determine the sensed methane concentrations [37]. Although both n-type and p-type MO semiconductors have been explored, n-type MOs act as the majority in methane sensors because oxygen vacancies are inevitably produced in MOs, serving as shallow donors to induce spontaneous electrons [38]. On the other hand, p-type MOs are beneficial for sensors with novel structures like heterostructures, improving sensor performance [39]. Therefore, this review aims to discuss the frontier advancements in gas sensors for CH_4_ and CO based on MO semiconductor materials, which are oriented toward coal mine safety [40].

### 1.1. Scope, Novelty, and Literature-Search Methodology

The novelty of this review is not intended to be a simple enumeration of metal-oxide methane and carbon-monoxide sensors. Instead, the literature is reorganized around the practical question faced by mine-safety researchers as shown in Table 2: Which sensing strategy offers the most appropriate balance among detection capability, selectivity, response/recovery speed, operating temperature, power consumption, stability, and compatibility with underground deployment? Particular emphasis is therefore placed on (i) the different surface-reaction requirements of CH_4_ and CO, (ii) quantitative comparisons of representative sensor architectures, (iii) selectivity and simultaneous CH_4_/CO detection, and (iv) the environmental and safety constraints of coal mines. This reader-oriented framework distinguishes the present review from reviews that focus primarily on material synthesis or on a single target gas.

### 1.2. Literature-Search Methodology

This review was updated by screening the literature cited in the original manuscript and by incorporating recent metal-oxide gas-sensing studies identified in the reviewer-suggested literature. The search was organized around combinations of the terms “metal oxide semiconductor”, “chemiresistive gas sensor”, “methane/CH_4_”, “carbon monoxide/CO”, “coal mine”, “selectivity”, “humidity”, and “room temperature”. Studies were retained when they provided experimental or mechanistic information relevant to metal-oxide semiconductor sensing of CH_4_ or CO. DFT papers were retained only when they directly support the interpretation of an experimentally relevant sensing mechanism; purely theoretical studies are not treated as independent sensor demonstrations. Optical SPR and catalytic-pellistor studies are not included as core chemiresistive MOS results and are discussed only for comparisons or application contexts. The search emphasizes recent developments while retaining foundational studies needed to explain sensing mechanisms. The recent literature used to update the review includes a 2026 review of semiconductor metal-oxide methane sensors and a 2025 review of MOS-based gas sensors.

Definition of “Recent Advancements”: In this review, “recent” refers primarily to developments published during approximately the last five years, while older papers are retained when they establish a mechanism, benchmark a material, or provide an important integration concept. Consequently, this review is chronological only where necessary and is primarily organized by sensing material, mechanism, and practical function.

**Table 2:** Mine-oriented performance requirements and practical implications.

Parameter	Mine-Safety Implication	Interpretation for MOS Sensors
CH_4_ concentration	Warning must precede the flammability/explosion regime; the manuscript cites 1% for warning, 1.5% for mandatory evacuation, and 5–15% as the flammable range.	ppm-level laboratory detection is useful for early warning, but sensitivity alone is not sufficient; the sensor must remain reliable over the relevant %–ppm transition range and under changing humidity/oxygen.
CO concentration	The manuscript cites 24 ppm for warning (spontaneous combustion) and 50 ppm for mandatory evacuation action thresholds.	A sensor demonstrating a low ppm or sub-ppm LOD is not automatically better if selectivity, drift, humidity tolerance, or calibration stability is poor.
Selectivity	CH_4_ and CO can coexist, and other mine gases can interfere.	A practical system should report discrimination capability, not only single-gas sensitivity.
Response/recovery	Rapid warning is essential during transient gas accumulation or fire development.	Short response is valuable, but must be evaluated together with recovery, baseline drift, and repeated-cycle stability.
Operating temperature/power	Heated sensors may increase power demand and introduce a safety concern in methane-containing atmospheres.	Low-temperature, light-activated, or microheater/MEMS approaches are attractive when they preserve sensitivity and stability.
Environmental robustness	Humidity, oxygen concentration, dust, condensation, pressure, ventilation, and vibration vary underground.	Performance reported only under dry laboratory air should not be treated as equivalent to field readiness.

The key practical point is that “ppm sensitivity” should not be interpreted as a stand-alone criterion. The manuscript cites 1% CH_4_ (alarm), 1.5% CH_4_ (action), 24 ppm CO (warning), and 50 ppm CO (action) as important mine-safety boundaries; therefore, a useful sensor must provide an adequate margin below the relevant alarm/action concentration while also maintaining selectivity, response speed, calibration stability, and environmental robustness. For CH_4_ in particular, the difference between a low laboratory detection limit and reliable operation across the mine-relevant concentration range must be made explicit. Thus, the best sensing approach is defined here by a multi-parameter performance envelope rather than by the lowest reported detection limit.

## 2. Sensing Mechanism and Improvement Methods

### 2.1. MO Reduction Gas-Sensing Mechanism

Before any target reducing gas is introduced, the MO sensor is usually brought to an elevated operating temperature, typically ranging from 150 °C to 450 °C [41]. This thermal activation is essential because it provides the required activation energy for both the adsorption of ambient oxygen and the subsequent chemical reactions with reducing gases [42].

When the sensor is exposed to normal atmospheric air at these high temperatures, ambient oxygen molecules (O_2_) adsorb onto the exposed surfaces, steps, and kinks of the metal oxide polycrystalline grains [43]. Because metal oxides have a high affinity for electrons, these adsorbed oxygen molecules do not remain neutral; instead, they act as extrinsic surface acceptor states and capture free electrons from the conduction band of the bulk semiconductor material [44]. Depending on the operating temperature, the oxygen undergoes a series of chemisorption transitions:

$$O_{2}\left(gas\right)\to O_{2}\left(adsorbed\right)$$

$$O_{2}\left(adsorbed\right)+e^{-}\to O_{2}^{-}\;(<150\;{}^{\circ}C)$$

$$O_{2}^{-}+e^{-}\to {2O}_{2}^{-}\;(150\;{}^{\circ}C-30\;{}^{\circ}C)$$

$$O^{-}+e^{-}\to O^{2-}\;(>300\;{}^{\circ}C)$$

For clarity, the oxygen-adsorption sequence is written in a temperature-dependent form rather than as a single universal sequence. A commonly used representation is

O_2_(gas) + e^−^ → O_2_*^−^*(adsorbed)

O_2_^−^(adsorbed) + e^−^ → 2O^−^(adsorbed)

O^−^(adsorbed) + e^−^ → O_2_^−^(lattice/adsorbed)

The last expression is deliberately qualified: Conversion among O^2−^, O^−^ and other oxygen species depends on the oxide surface and temperature, and the formation of atomic oxygen should not be written as an unconditional conversion of superoxide. The exact dominant species should therefore be supported by the material-specific literature.

In most commercial MO sensors operating at optimal performance temperatures, the dominant chemisorbed species are the ionic forms O^−^ and O^2−^. The immobilization of these free electrons at the surface profoundly alters the electronic structure of the material. For an n-type semiconductor (such as tin dioxide or zinc oxide), where electrons are the majority charge carriers, this trapping effect severely depletes the surface region of its mobile electrons. This leads to the formation of a space-charge region known as the electron depletion layer (EDL) [45].

As electrons are pulled to the surface, the local electrostatic potential changes, inducing an upward bending of the energy bands at the grain boundaries [42]. This creates an intergranular potential barrier that can strongly control carrier transport. The term “Schottky barrier” is reserved here for interfaces for which a true metal–semiconductor Schottky junction is established; an ordinary semiconductor–semiconductor grain boundary is more appropriately described as an intergranular potential barrier or grain-boundary barrier [44]. Because electrons must overcome this energy barrier to flow through the material, the formation of the EDL results in a highly resistive baseline state in ambient air. The resistance of the sensor in air (R_a_) is thus kept deliberately high. When a reducing gas like carbon monoxide (CO) or methane (CH_4_) enters the sensor environment, it actively interacts with the highly sensitive MO surface at the grain boundaries and necks. Because these target gases are chemically reducing, they serve as fuel for an oxidation reaction with the pre-adsorbed, highly reactive oxygen anions (O^−^ or O^2−^).

(1) CH_4_ Detection

**Figure 2:**
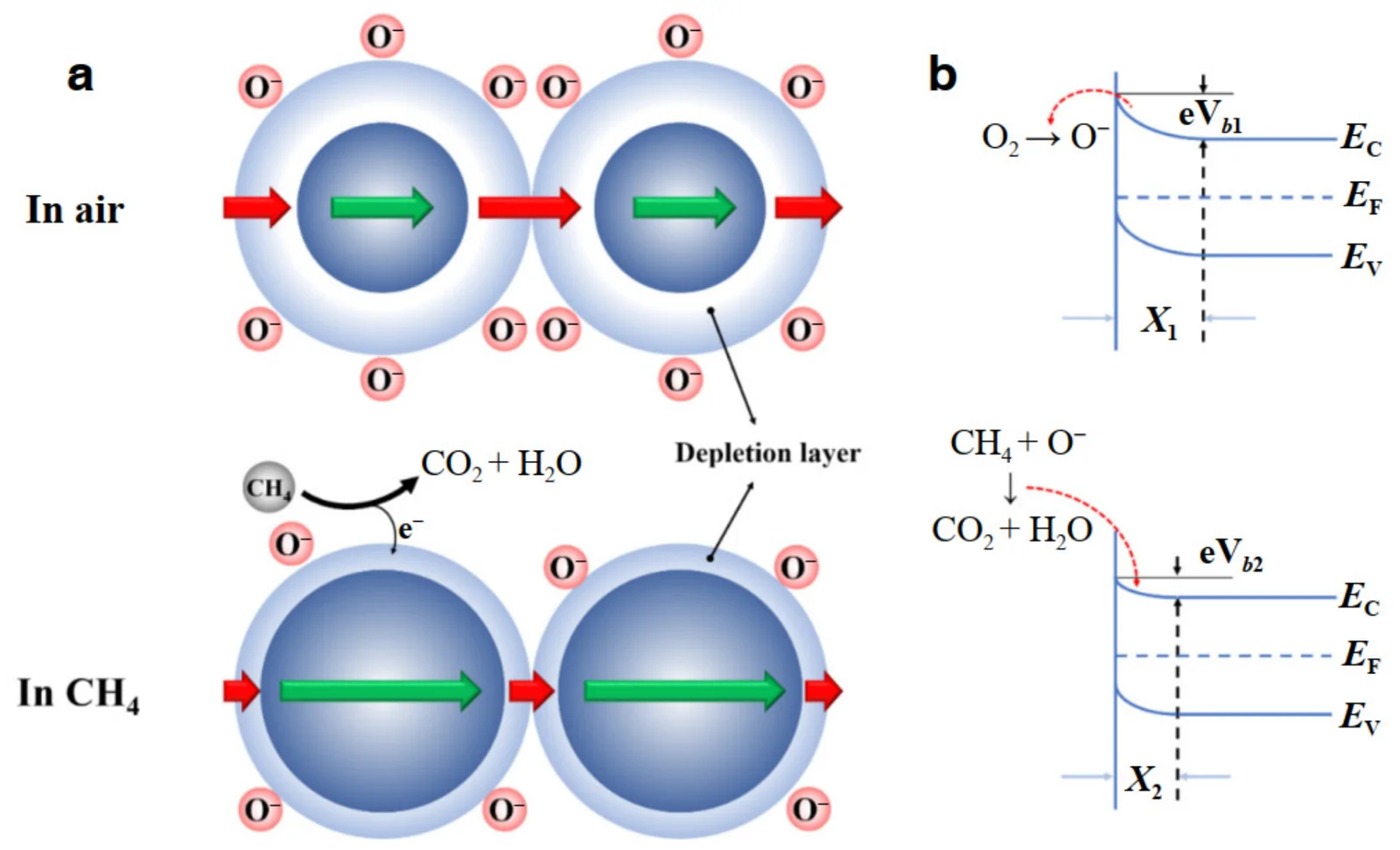
Schematic illustration of n-type MO in CH_4_ sensing: (**a**) electron depletion layer and (**b**) corresponding energy band diagram. Reproduced from Reference [43], with permission from INOE Publishing.

As displayed in Figure 2, methane sensing is intrinsically limited by the high chemical stability of the CH_4_ molecule, which originates from its strong tetrahedral C–H bonds (bond energy approximately 439 kJ mol^−1^). Therefore, methane oxidation requires an initial catalytic activation step involving C–H bond cleavage, followed by successive oxidation reactions with surface oxygen species to generate CO_2_ and H_2_O while releasing electrons back into the semiconductor. This process strongly depends on the catalytic activity of the sensing material, surface oxygen mobility, and operating temperature, explaining why methane sensors typically require elevated working temperatures to achieve sufficient response [46,47]. However, once activated, the mechanism follows a similar oxidative path, breaking down into carbon dioxide and water vapor [46,48]:

$${CH}_{4}\left(gas\right)+4O_{ad}^{-}\left(surface\right)\to {CO}_{2}\left(gas\right)+2H_{2}O+4e^{-}$$

During this reaction, the methane molecule combines with the oxygen ion to form carbon dioxide (CO_2_) and water (H_2_O), which safely desorbs back into the surrounding atmosphere. Crucially, the electron that was originally trapped by the oxygen molecule is released and injected back into the conduction band of the semiconductor [48].

(2) CO Detection

**Figure 3:**
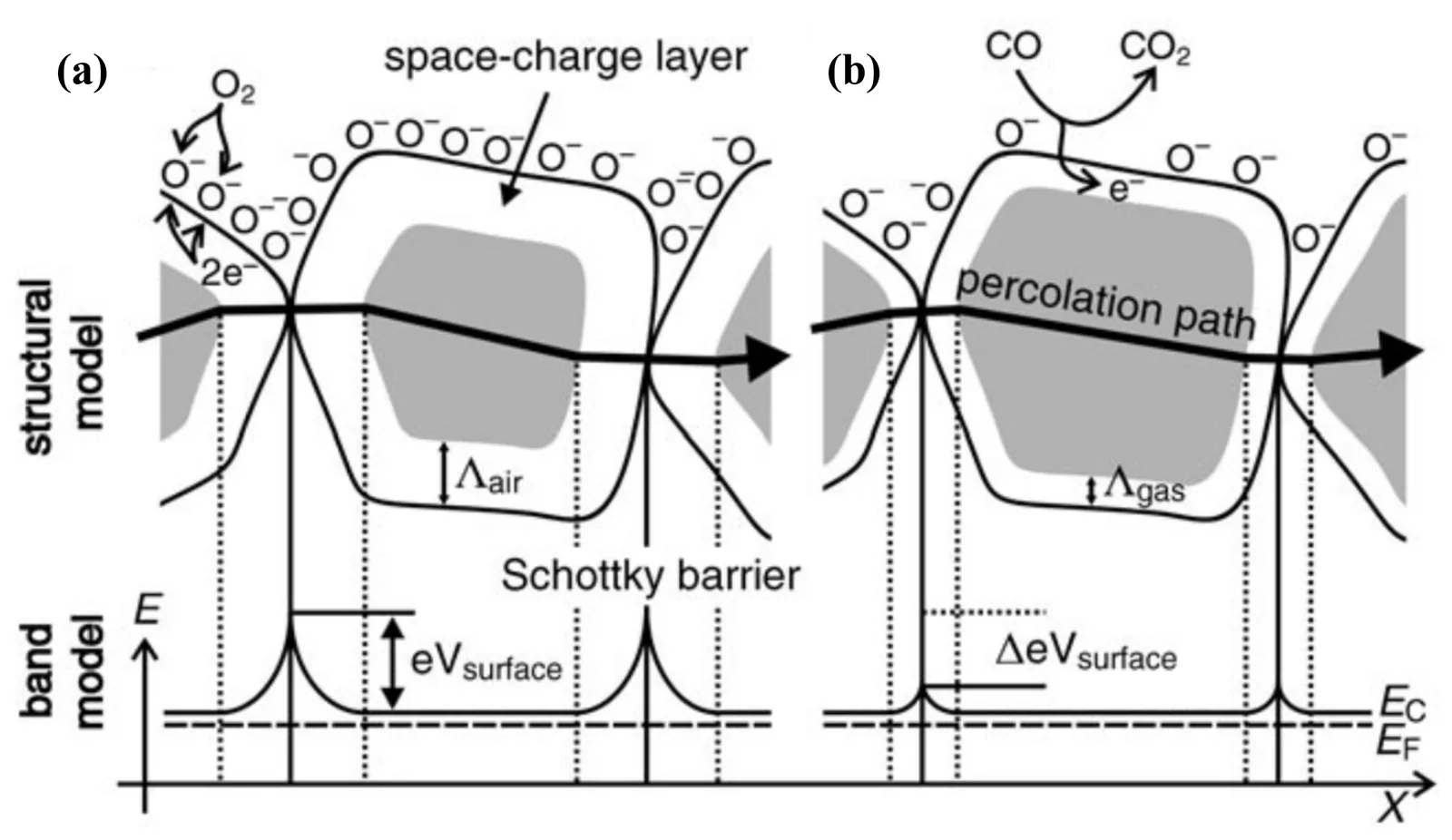
Structural and band model showing the role of intergranular contact regions in determining the conductance over a polycrystalline metal oxide semiconductor: (**a**) initial state and (**b**) effect of CO for large grains. Reproduced from Reference [49], with permission from Wiley-VCH.

Carbon monoxide exhibits significantly higher surface reactivity than methane because it does not require preliminary bond-breaking activation. CO molecules can directly participate in catalytic oxidation reactions with chemisorbed oxygen ions on the metal oxide surface, producing CO_2_ and releasing electrons into the conduction band. As a result, CO sensing generally proceeds with faster reaction kinetics and lower activation barriers compared with methane detection (Figure 3). Consequently, while both gases induce resistance modulation through oxygen-mediated redox reactions, methane detection is primarily governed by the ability of the sensing surface to activate stable C–H bonds, whereas CO detection is controlled by the rapid oxidation of an already reactive reducing molecule [49,50,51]. The chemical equation representing the interaction of CO is

$$CO\left(gas\right)+O_{ad}^{-}\left(surface\right)\to {CO}_{2}\left(gas\right)+e^{-}$$

Just like CH_4_, the oxidation of carbon monoxide liberates a significant number of trapped electrons, returning them directly to the bulk semiconductor matrix [51,52].

The return of these trapped electrons back into the conduction band instantly triggers a massive electronic shift within the material [53]. As the electron concentration in the conduction band surges, the thickness of the EDL shrinks dramatically [53,54]. Consequently, the upward energy band bending relaxes, and the height of the potential barrier between adjacent crystalline grains drops [54]. With the electronic roadblocks cleared, electrons can easily flow across the percolation paths throughout the polycrystalline network.

In a p-type MO semiconductor, oxygen adsorption withdraws electrons from the oxide and increases the near-surface hole concentration, producing a hole-accumulation layer (HAL). Exposure to a reducing gas returns electrons to the oxide; these electrons recombine with holes, reducing the HAL and increasing resistance. This opposite carrier response is useful for constructing complementary sensing elements, but it does not by itself solve CH_4_/CO discrimination because both gases are reducing gases.

Therefore, for n-type MO Materials (SnO_2_, ZnO, WO_3_), the introduction of a reducing gas causes a measurable, sharp decrease in electrical resistance (R_g_ < R_a_) [55]. For p-type MO materials (CuO, NiO, and Co_3_O_4_), holes are the majority charge carriers, and the injection of electrons annihilates holes (recombination) [55,56]. This shrinks the hole accumulation layer, resulting in a measurable increase in electrical resistance [57]. By continuously measuring the ratio of the sensor resistance in air to its resistance in the target gas (for n-type, the response is often defined as S = R_a_/R_g_; for p-type, S = R_g_/R_a_, while many works report percentage change, S = (R_a_/R_g_) × 100% or S = (ΔR/R_a_) × 100%), the external circuitry can precisely quantify and display the concentration of the reducing gas in real time. It is important to note that the specific response definition varies across studies; a direct numerical comparison of response values from different works is not valid unless the same definition and test conditions are used.

### 2.2. MO Sensing Improvement Methods

(1) Metal Modification and Doping

Noble Metal Modification: Incorporating noble metals like silver (Ag), platinum (Pt), or gold (Au) significantly amplifies sensor capabilities due to their excellent catalytic properties. For instance, modifying MO with Ag nanoparticles introduces electronic and chemical sensitization mechanisms, forming Schottky junctions that exponentially enhance current signals and increase response values while reducing operating temperatures [58,59].

Transition and Rare Earth Metal Doping: Doping with transition metals such as nickel (Ni) or rare-earth elements like cerium (Ce) and lanthanum (La) optimizes surface defect concentrations, adjusts the bandgap, reduces contact potential, and accelerates charge transfer. Specifically, a smaller bandgap makes it easier for electrons to transition from the valence band to the conduction band [60,61,62].

(2) Morphological and Structural Engineering

Porous and Hierarchical Structures: Fabricating porous or hierarchical structures (e.g., mesoporous or porous microspheres) improves charge transfer, gas diffusion, and surface permeability [63]. This three-dimensional setup provides open channels for gas diffusion and increases the specific surface area, providing more active sites for target gas adsorption and reaction.

One-Dimensional (1D) Nanostructured Architectures: Designing 1D assembled nanostructures—such as tubes, rods, fibers, and belts—enhances the active surface area [64]. Using surface-engineered 1D structures (like nanobelts/nanoribbons) helps prevent the structures from packing too closely, ensuring better gas analyte diffusion into the internal structures compared to standard nanofibers [65,66].

(3) Surface Defect Engineering

Oxygen Vacancies: Adjusting material composition through doping (e.g., Ni doping) increases the concentration of surface oxygen vacancy defects. These oxygen vacancies act as vital adsorption sites during gas reactions, enabling more target gas molecules to interact with oxygen species on the sample surface, which boosts sensitivity, selectivity, and response values [67,68].

Annealing Regulation: The performance of the sensor is heavily influenced by post-synthesis thermal treatments. Optimizing the annealing temperature allows control over the material’s textural behavior, particle size, and defect density. For example, a proper annealing temperature yields small-sized, mesoporous particles with a high concentration of oxygen vacancies, significantly enhancing gas adsorption and desorption channels [69].

### 2.3. Critical Design Factors for Coal-Mine Deployment

Operating Temperature: The frequently cited 150–450 °C range describes many conventional thermally activated MOS sensors, not a universal requirement. The reviewed literature includes UV-assisted, organic–inorganic, 2D-material-assisted, and other low-temperature or room-temperature approaches. These strategies can reduce heater power and the thermal hazard in methane-containing atmospheres, but they may introduce dependence on illumination, humidity, surface contamination, or material stability. Therefore, low temperatures should be treated as a deployment advantage only when accompanied by adequate selectivity, repeatability, lifetime, and calibration stability.

Mine-Environment Effects. Underground sensors may experience temperature variation, high relative humidity, pressure variation, changing ventilation speed, coal dust, condensation, mechanical vibration, and exposure to chemically reactive species. Water can compete for adsorption sites and alter the surface hydroxyl population; dust and condensation can block active sites or gas-diffusion pathways; ventilation changes mass transfer and therefore apparent response time; long-term exposure can change the baseline through poisoning, grain growth, defect redistribution, or electrode degradation. These factors should be incorporated into validation protocols rather than discussed only as secondary limitations. Evidence from TiO_2_ nanowire sensors also shows that temperature and humidity can substantially alter gas response and baseline behavior [70].

Oxygen Dependence. Because MOS sensing relies on surface oxygen adsorption, changes in oxygen partial pressure can change the baseline resistance and the magnitude of the gas response. Oxygen partial pressure (P_O2_) governs the coverage of surface chemisorbed oxygen species (O_2_^−^, O^−^, and O^2−^), which directly determine baseline resistance (Rₐ) and gas response (Rₐ/R_9_ for n-type). In poorly ventilated mine zones where P_O2_ can drop below 15%, several effects occur:

(1) Lower Baseline Resistance—Fewer adsorbed oxygen anions withdraw fewer electrons, thinning the electron depletion layer and reducing Rₐ, which shrinks the dynamic range for gas detection.

(2) Reduced Response Magnitude—Less surface oxygen limits oxidation of CH_4_ and CO, especially for methane, for which its C–H activation already requires catalytic assistance.

(3) Slower Response/Recovery Kinetics—Oxygen replenishment during recovery is hindered, prolonging recovery times.

(4) Shifted Optimal Operating Temperature—The temperature at which maximum response occurs changes with P_O2_, complicating calibration.

Poorly ventilated or oxygen-depleted mine regions therefore cannot necessarily be represented by calibration data collected in ordinary laboratory air. A practical sensor should characterize response versus oxygen concentration or include compensation through reference sensors and system-level calibration.

Selectivity and Simultaneous CH_4_/CO Sensing: CH_4_ and CO are both reducing gases and can drive the resistance of an n-type MOS in the same direction. Consequently, a single unmodified MOS resistance measurement generally cannot identify which gas caused the signal. Practical discrimination requires chemically selective catalysts or filters, different operating temperatures, multiple sensing elements with complementary materials, p-/n-type arrays, modulation by light or temperature, or pattern-recognition/electronic-nose and machine-learning approaches. Mine-relevant interferents include H_2_, H_2_S, SO_2_, NO_x_, CO_2_, NH_3_, hydrocarbons, and VOCs. Selectivity should also be tested at realistic concentration ratios rather than only by exposing the sensor to equal concentrations of each gas. Electronic-nose and pattern-recognition approaches provide a relevant model for discrimination in multi-component gas environments.

Safety and Integration: A sensor intended for methane-containing atmospheres must minimize ignition risk and power consumption. MEMS microheaters can reduce thermal mass and energy demand, while CMOS-compatible fabrication can support compact readout and multi-sensor arrays. However, integration should be evaluated together with packaging, thermal isolation, electromagnetic robustness, dust/condensation protection, calibration, wireless communication, and explosion-proof requirements; laboratory integration alone does not demonstrate mine readiness.

## 3. MO Methane Gas Sensors

### 3.1. n-Type MO

(1) Indium oxide (In_2_O_3_)

In_2_O_3_ has been shown to possess surface-rich defects (e.g., oxygen vacancies) and high electrical conductance, contributing to its huge success in electronic devices, like thin-film transistors and memory. In addition to its excellent chemical stability, attention has been focused on indium oxide with respect to widespread gas sensors, for example, methane. N. M. Shaalan et al. demonstrated a methane sensor with indium oxide via a handy thermal evaporation method. A high transmittance of 96% and a wide bandgap of 3.68 eV were concurrently obtained in the sensor. It can detect a methane concentration of 0.25%, which is much lower than the explosive concentration (5%), supporting effective warning [30].

**Figure 4:**
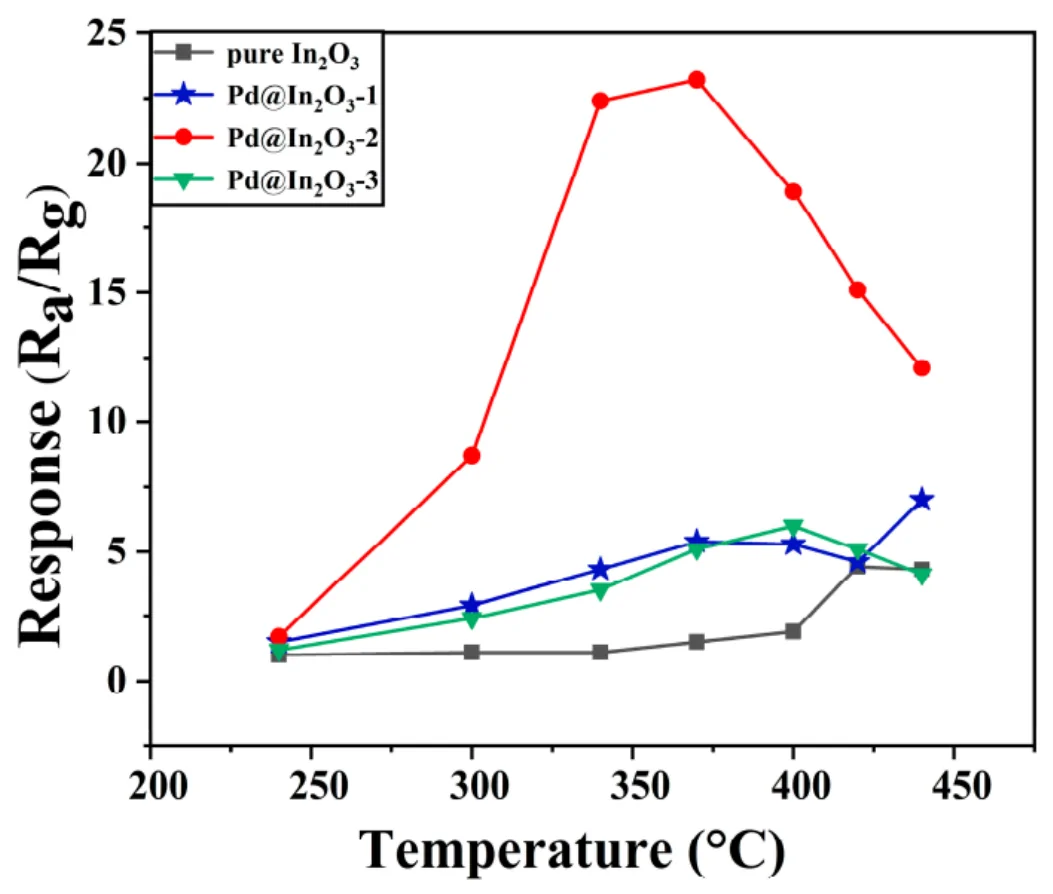
Gas response of pristine In_2_O_3_, Pd@In_2_O_3_-1, Pd@In_2_O_3_-2, and Pd@In_2_O_3_-3 to 5000 ppm CH_4_ at different temperatures. Reproduced from Reference [72], with permission from CC BY 4.0.

However, the sensitivity and selectivity of pure In_2_O_3_ are limited, especially at low temperatures. One efficient way to improve methane-sensing response is to artificially construct porous nanostructures, which offer a large specific surface area and surface-to-volume ratio for more active reactions of methane molecules. The hydrothermal method is a handy way to obtain nanostructures, such as nanosheets, nanospheres, nanocubes, and nanowires. D. Xue et al. synthesized porous In_2_O_3_ nanosheets via a one-step hydrothermal method, producing a cubic structure of In_2_O_3_ without impurity peaks, as evidenced by XRD measurements. Importantly, the pore size of the In_2_O_3_ samples is mainly around 3.3 nm, with the maximum size smaller than 25 nm, reflecting high mesoporous properties with a 96 m^2^g^−1^ specific surface area. As a result, the methane sensor illustrates a high response at 190 °C operation temperature and strong stability, as reflected by marginal degradation for sensing 500 ppm CH_4_ over 30 days [71]. X. Zuo et al. successfully synthesized Pd nanoparticles embedded in In_2_O_3_ porous hollow tubes (Pd@In_2_O_3_ PHTs) using Pd@MIL-68(In) MOFs as precursors via a solvothermal method combined with calcination and H_2_ reduction (Figure 4). XRD patterns confirmed the cubic In_2_O_3_ structure with no detectable Pd peaks due to high dispersion. TEM and HRTEM revealed hexagonal hollow tubes with abundant pores and lattice fringes of Pd (111) and In_2_O_3_ (211), while XPS and EPR indicated increased oxygen vacancies (31.01%) and a narrow band gap (2.50 eV). As a result, the Pd@In_2_O_3_-2 sensor exhibited a high response of 23.2 to 5000 ppm CH_4_ at 370 °C (15.5 times that of pristine In_2_O_3_), with rapid response/recovery times (7 s/5 s) and excellent selectivity and stability over 30 days [72]. Interestingly, belt-like In_2_O_3_ with mesoporous morphology was developed by M. B. Kgomo et al. via a single-step electrospinning method. Combined with post-annealing treatment, a response of 1.1 to 90 ppm of methane at a lower operating temperature of 100 °C is achieved in the sensor, which demonstrates fast response and recovery times of 36 and 44 s, respectively [73].

Another important pathway to boost the sensing performance is functional modification, e.g., metal doping, which can enhance surface ion adsorption with modulated surface defects. Y. Zhang et al. adopted nickel as the dopant in indium oxide due to the smaller radii of Ni^2+^ than In^3+^ ions, which can easily adjust bandgap and defects. Various molar ratios of Ni/In have been investigated to optimize the device performance, and an improved response of 72.727 to 200 ppm CH_4_ is obtained [74]. Meanwhile, this group also demonstrated a high-performance methane sensor adopting Ag-doped In_2_O_3_ with porous microspheres [75]. As a result, a lower operation temperature of 120 °C is achieved with a 27.5 response to 500 ppm methane, which is improved by over 100% compared to pristine In_2_O_3_. O_2_ can be dissociated into oxygen atoms that can spill onto the In_2_O_3_ surface to combine with electrons from its conduction band, generating more oxygen ions (e.g., O^−^). Meanwhile, the dissociation of methane can also be facilitated into CH_3_ and H to react with oxygen ions, dramatically reducing the resistance of Ag/In_2_O_3_.

(2) Tin oxide (SnO_2_)

Tin oxide (SnO_2_) is another famous MO semiconductor that has been widely explored for chemical gas sensors due to its ease in processing and desirable response to a number of gases at low operation temperatures. One crucial obstacle for high-performance SnO_2_ sensors is weak selectivity to various gases. Moreover, challenges with respect to poor stability, low gas response, and high operating temperatures still exist and hinder the extensive application of sensors. One effective way is to utilize metallic particles as catalysts to enhance the sensing response of SnO_2_ for reducing gases like methane. D. Haridas et al. investigate methane gas detection utilizing rf-sputtered SnO_2_ thin films functionalized with nanoscale catalytic clusters [76]. By systematically loading ultrathin layers of various catalysts (Pt, Ag, Ni, Pd, Au, NiO, and Au_2_O_3_), the authors demonstrate that the SnO_2_-Pd cluster configuration results in optimized performance. The sensor exhibits a highly enhanced response of 97.2% to 200 ppm of methane at a lowered operating temperature of 220 °C. This pronounced electronic improvement is primarily attributed to the spill-over mechanism of Pd clusters, which lowers the activation energy for methane dissociation and accelerates surface reactions.

On the other hand, noble metal catalysts applied should be extremely thin to avoid short-circuiting through the sensor, and metals can diffuse into the sensor material, especially at high working temperatures. Recently, SnO_2_ decorated by other MOs has been extensively developed to support high-performance SnO_2_ gas sensors. D. Xue et al. demonstrated a 3D hierarchical WO_3_-SnO_2_ nanoflower composite synthesized via a facile impregnation method to optimize MO methane sensors [77]. The unique structure consists of small WO_3_ nanoplates systematically dispersed across the surfaces of larger, self-assembled SnO_2_ nanoflowers. Gas-sensing evaluations reveal that the WO_3_/SnO_2_ composite significantly outperforms pure SnO_2_. It reduces the optimum operating temperature from 120 °C to 110 °C and exhibits a response to 500 ppm of methane that is 2.3 times higher than its pristine counterpart. Furthermore, the sensor demonstrates a remarkably low limit of detection (38 ppb), alongside high stability and repeatability. This performance boost is primarily credited to an expanded specific surface area and the synergistic formation of electronic n-n heterojunctions at the materials’ interface.

**Figure 5:**
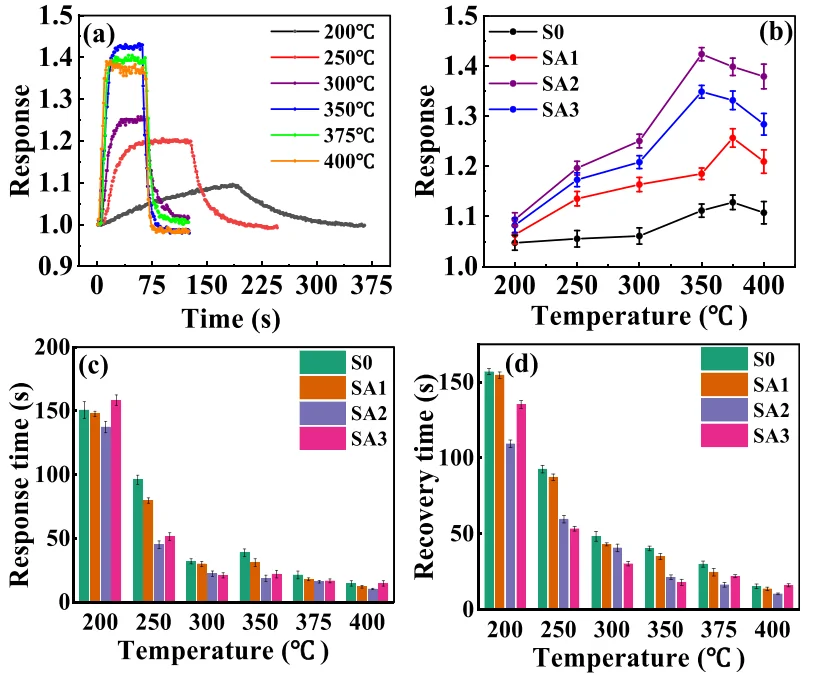
Response characteristics of different SA sensors to CH_4_. (**a**) Response curves of the SA_2_ sensor toward 2000 ppm CH_4_ at different operating temperatures. (**b**) Response of SA sensors toward 2000 ppm CH_4_ at different operating temperatures. (**c**) Response time of SA sensors toward 2000 ppm CH_4_ at different operating temperatures. (**d**) Recovery time of SA sensors toward 2000 ppm CH_4_ at different operating temperatures. The results in (**b**–**d**) are averaged from three independent measurements, and the error bars show the corresponding standard deviations. Reproduced from Reference [78] with permission from CC BY 4.0.

Interestingly, M. Jiao et al. successfully fabricated SnO_2_-Ag-ZnO composite methane sensors via magnetron sputtering [78]. As shown in Figure 5, Ag doping reduced the optimal operating temperature and enhanced the response by 1.79-fold compared to pure SnO_2_, while ZnO’s introduction further amplified gas adsorption through n-n heterojunction effects. SEM and XPS confirmed uniform elemental distribution and increased oxygen vacancies. The SAZ2 sensor achieved a response of 2.03 to 2000 ppm CH_4_ at 350 °C with rapid response/recovery times (10 s/8 s) and a low detection limit of 33 ppm. Moreover, integrating a SqueezeNet transfer learning model enabled 91.6% classification accuracy for combustible gas mixtures, demonstrating robust selectivity in complex environments.

(3) Zinc oxide (ZnO)

ZnO has been extensively explored as one of the earliest sensing oxides for gas sensors due to its high electron mobility and reproducibility, photoelectric response, and thermal stability. However, the operating temperature of the ZnO gas methane sensor is relatively high, along with limited response, so X. Sun et al. developed a room-temperature ZnO methane sensor via UV illumination [79]. ZnO rods, plates, and spheres were prepared to fabricate methane sensors, all of which can clearly respond to methane gas under UV illumination but hardly respond in the dark. Among the three structures, the ZnO sphere device demonstrates the highest response and the shortest response time, which is experimentally and theoretically attributed to the hollow structure and more Zn Atoms as active sites on the exposed facet of (001).

**Figure 6:**
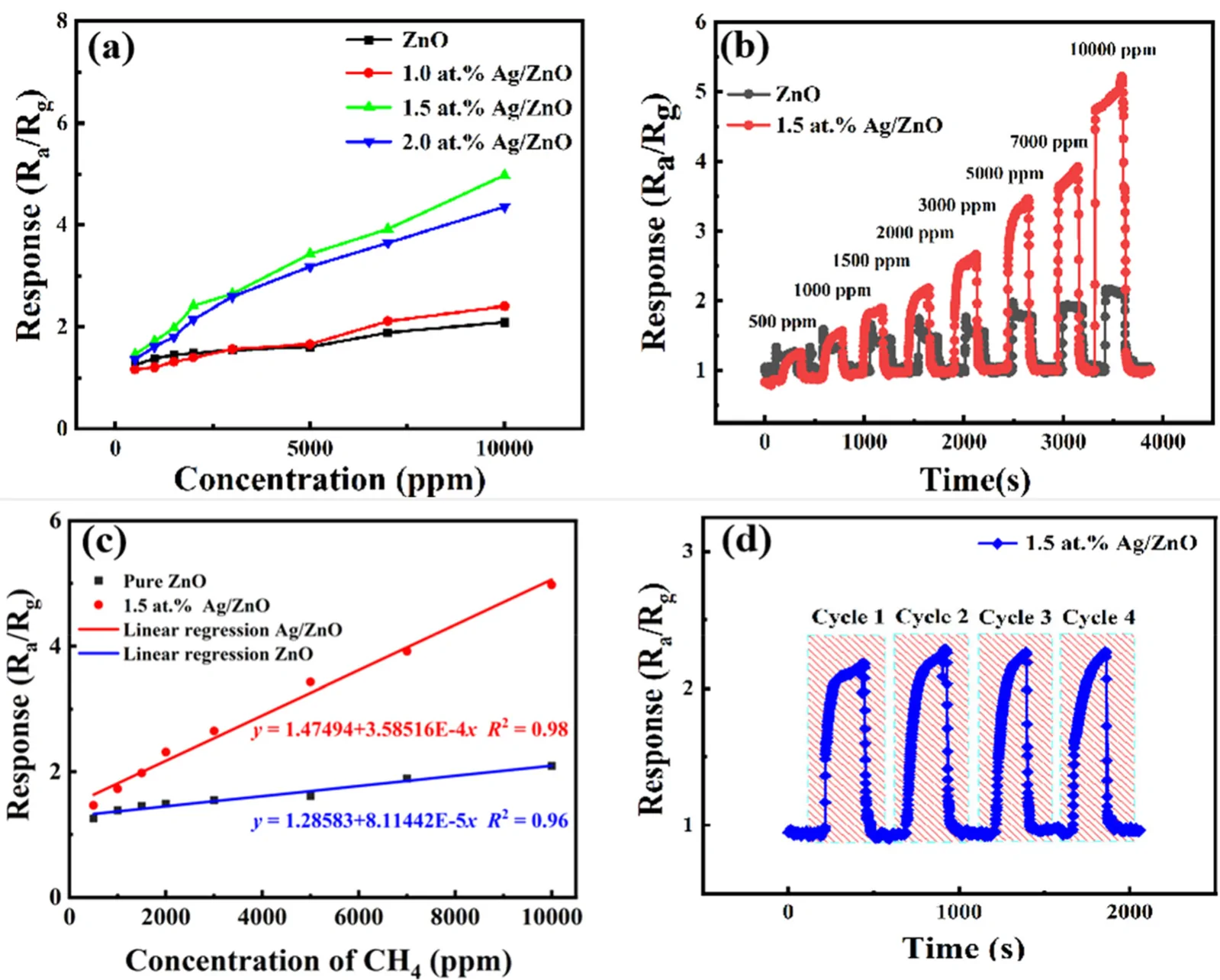
(**a**) Response of samples with different concentrations of Ag to all methane concentrations. (**b**) Transient response curves of ZnO and 1.5 at% Ag/ZnO at room temperature, (**c**) their corresponding fitting curves, and (**d**) repeatability tests toward 2000 ppm CH4 of the 1.5 at Ag/ZnO sensor at room temperature. Reproduced from Reference [81], with permission from CC-BY-NC 4.0.

Moreover, surface decorations, including organic and inorganic ones, are also widely utilized in ZnO methane gas sensors to improve sensing performance. T. Sen et al. successfully developed polyaniline/zinc oxide (PANI/ZnO) nanoparticles as a room-temperature sensing layer for methane [80]. As shown in Figure 6, M. Li et al. successfully synthesized Ag-modified flower-like ZnO microspheres via a solvothermal method combined with an impregnation process [81]. XRD patterns confirmed hexagonal wurtzite ZnO structures with no detectable Ag peaks due to low loading and uniform dispersion. FESEM and TEM revealed hierarchical porous microspheres assembled from nanosheets, with Ag nanoparticles (3–5 nm) evenly distributed on ZnO surfaces, while XPS and UV–vis indicated increased oxygen vacancies and enhanced visible-light absorption with a narrowed band gap (3.10 eV). As a result, the 1.5 at% Ag/ZnO sensor exhibited a superior response of 3.43 to 5000 ppm CH_4_ at room temperature under simulated sunlight, ~206% higher than pristine ZnO, with fast response/recovery times (47 s/35 s) and good repeatability. The enhanced performance is attributed to the local surface plasmon resonance (LSPR) and catalytic effects of Ag nanoparticles.

Additionally, M. Gul et al. doped ZnO with magnesium for methane detection [82]. This study details the synthesis of magnesium (Mg)-doped zinc oxide (ZnO) nanostructures via a vapor transport method on silicon substrates. Doping transformed the morphology from undoped ZnO nanorods to crystalline wurtzite Mg-doped nanobelts. Optical analyses revealed a widened energy bandgap from 3.18 eV (undoped) to 3.32 eV (doped), attributed to the Burstein–Moss effect. When tested as sensors, the Mg-doped nanobelts exhibited a significantly enhanced photocurrent (189 μA) and excellent UV sensing response. Crucially, the doped nanobelts achieved a prominent 54% sensing response toward 400 ppm of methane gas at an optimal temperature of 200 °C. This performance improvement is driven by an expanded surface area, increased active oxygen vacancies, and accelerated gas adsorption–desorption kinetics introduced by the Mg dopant.

Interestingly, Bhattacharyya et al. exploited microelectromechanical technology to fabricate a low-power microelectromechanical system (MEMS) methane gas sensor based on nanocrystalline zinc oxide (ZnO) thin films, which was integrated onto a micromachined silicon substrate [83]. The device demonstrates high compatibility with standard CMOS integration. Optimized at an operating temperature of 250 °C, the sensor achieves a high response magnitude (87.3%) alongside rapid response (8.3 s) and recovery (17.8 s) times when exposed to 1.0% methane, consuming only 120 mW of power. Crucially, the sensor retains an appreciable response (44.5%) even at a low operating temperature of 100°C, drawing a minimal 43 mW.

(4) Tungsten Oxide (WO_3_)

Jaroenapibal et al. systematically evaluated the methane sensing behavior of electrospun tungsten oxide WO_3_ nanofibers through impedance spectroscopy [84]. By modulating precursor concentrations and calcination parameters, monoclinic WO_3_ nanofibers with controlled internal particle heterogeneities ranging from 29 to 100 nm were successfully synthesized. Crucially, the authors unveil that optimal operating temperatures are inversely proportional to internal particle size. The finest nanostructure (29 nm) lowers the optimal operating temperature to 200 °C while achieving the highest sensitivity (S = 2.85) to 1000 ppm of methane. Complex Nyquist plots reveal that smaller grains dramatically diminish grain-boundary relaxation times. This enhanced performance is governed by morphology-induced catalytic pathways that lower target-gas activation energy and accelerate overall response dynamics.

Feng-Chen et al. reported highly sensitive prototypic methane gas sensors built from nanostructured tungsten oxide composite nanowires [85]. Synthesized via hot-filament chemical vapor deposition (CVD), each nanowire comprises crystalline nanoparticles with diameters under 250 nm. Material analysis via XRD and Raman spectroscopy confirmed a monoclinic structure dominating in mixed states of WO_2_, WO_3_, and metallic W. Operating at room temperature, the sensor exhibits dual-mode behavior: It undergoes a chemisorption-driven resistance increase when exposed to methane and a resistance drop when exposed to hydrogen. For 2 ppm concentrations, response and recovery times settle around 1.5 and 1 min, respectively, accelerating down to a few seconds at 10 ppm methane. This enhanced methane sensitivity is governed by a synergistic competitive mechanism between the oxygen adsorbed on the oxide surfaces and the heavily distributed metallic W phase.

(5) Vanadium Oxide (V_2_O_5_)

Vanadium oxide, especially pentoxide (V_2_O_5_), has emerged as a highly advantageous material for chemiresistive methane gas sensors due to its unique structural properties and low-temperature catalytic activity. Unlike traditional metal oxides such as SnO_2_ or ZnO that often require high operational temperatures to overcome the high bond dissociation energy of methane (413 kJ/mol), V_2_O_5_ possesses a multi-valent nature with a stable V^+5^ oxidation state that acts as a classic heterogeneous catalyst to lower thermal barriers [86].

Mounasamy et al. successfully developed a high-performance, chemiresistive methane gas sensor by synthesizing hierarchical vanadium pentoxide thin films using DC magnetron sputtering [86]. By strategically tuning the sputtering power from 100 W to 150 W, the morphology of V_2_O_5_ can transform from flat nanorods to nano-urchins and finally into well-defined 3D nanoflowers composed of intertwined 2D nanosheets. XPS and GIXRD measurements reflect a stable, polycrystalline orthorhombic phase dominated by the reactive V^+5^ oxidation state. Strikingly, the V_2_O_5_ nanoflower sensor overcomes the traditional high thermal activation barriers of methane, achieving a distinct response of 8% toward 50 ppm of methane at a remarkably low operating temperature of just 100 °C without requiring noble metal catalysts. This optimized performance is governed by the high specific surface area, expanded slit-shaped internal voids, and an abundance of oriented surface active sites inherent to the hierarchical flower-like architecture, which altogether accelerate gas adsorption–desorption kinetics.

Baladeh and Haratizadeh present a compelling study on the low-temperature detection of methane utilizing hydrothermally synthesized V_2_O_5_ orthorhombic nanorods [87]. XRD and Raman results confirm well-crystallized, high-purity nanorods with an average crystallite size of 30 nm and an optical bandgap of 2.28 eV. While showing limited baseline reactivity at room temperature, the resistive sensor exhibits superior selectivity and sensitivity at a low temperature of 50 °C, with a 23% response at 4000 ppm and rapid response dynamics. Interestingly, the authors highlight a temperature-driven conduction transition from n-type to p-type. This surface-confined HAL inversion effectively addresses elevated-temperature safety hazards, rendering these nanorods highly promising for practical, low-temperature methane monitoring systems.

J. Liang et al. demonstrate a breakthrough in room-temperature methane detection utilizing Au-decorated vanadium oxide composite thin films [88]. Via dc-magnetron sputtering and rapid thermal annealing at 480 °C, the films develop a porous, cracked morphology dominated by an orthorhombic V_2_O_5_ phase. The sensor yielded its maximum response at room temperature toward a concentration of 1500 ppm of methane, demonstrating sharp performance degradation at elevated temperatures due to metal-insulator transitions. Sensor enhancement is driven by a high specific surface area, extensive oxygen vacancies, and a spill-over catalytic effect derived from Schottky contacts between the Au nanoparticles and the VO_x_ matrix. Moreover, they also successfully fabricated platinum (Pt)-decorated V_2_O_5_ for a methane gas sensor, which achieves a peak response of 18.2 toward 500 ppm of methane at room temperature [89].

Prasad et al. reported the first synthesis of novel single-phase, nanostructured vanadium dioxide (VO_2_) thin films specifically optimized for methane gas sensing [90]. The films are fabricated using a two-step approach consisting of pulsed dc-magnetron sputtering of a vanadium target followed by controlled oxidation at 550 °C. Characterization via GIXRD, FESEM, and HRTEM confirms a polycrystalline rutile monoclinic structure characterized by bundled nanorod architectures. The material exhibits a sharp, reversible semiconductor-to-metal transition (SMT) at roughly 68 °C. Crucially, gas-sensing experiments demonstrate that the films achieve a selective response to 50–500 ppm of CH_4_ at operating temperatures as low as 50 °C while remaining in their semiconducting state, offering a promising solution for near-room-temperature methane detection.

### 3.2. p-Type MO

(1) Nickel Oxide (NiO)

NiO has been proven to possess excellent oxygen adsorption, redox activity, and durability, which is ideal for hazardous gas monitoring, especially for methane. Their simple synthesis and superior sensing make NiO-based coral-like nanochains suitable for methane sensors.

**Figure 7:**
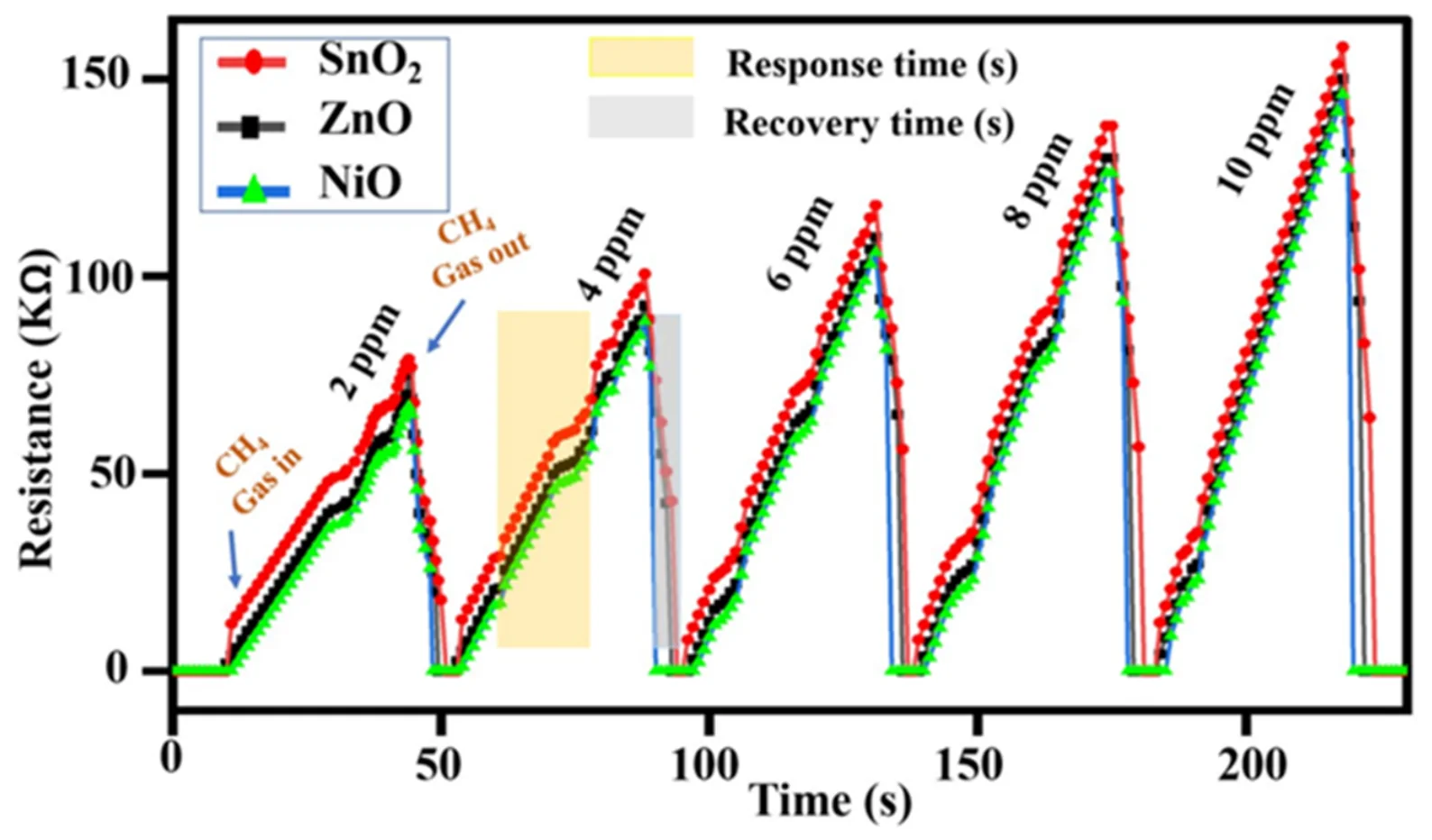
Gas sensors based on NiO nanostructures exhibit dynamic response and recovery behavior when exposed to CH_4_ gases at ambient temperature. Reproduced from Reference [91], with permission from CC-BY-NC 4.0.

As illustrated in Figure 7, P. Chellamuthu et al. synthesized CTAB-assisted NiO nanostructures via a hydrothermal method and integrated them with PEDOT:PSS to form a p-p heterojunction [91]. XRD confirmed a face-centered cubic NiO phase with a crystallite size of 4.43 nm and moderate micro-strain (0.013). FESEM revealed aggregated nanoparticles with rough porous surfaces, while BET analysis gave a specific surface area of 27.43 m^2^/g and a pore diameter of 9.4 nm. EIS indicated the highest charge-transfer resistance (2.1 kΩ) among the three oxides, reflecting limited conductivity. At room temperature, the PEDOT:PSS/NiO sensor showed sensitivities of approximately 62% for CH_4_ at 10 ppm, with response/recovery times of 20 s/12 s and 18 s/10 s, respectively—the lowest performance among the tested materials, attributed to its p-type conduction and fewer active sites.

E. Gagaoudakis et al. investigated transparent p-type aluminum-doped nickel oxide (NiO:Al) thin films for room-temperature methane gas detection [92]. Radio frequency (rf) magnetron sputtering is utilized to obtain a 10% Al doping concentration, and the 100 nm films exhibit high optical transparency (~80%) and an amorphous or poorly crystalline microstructure. Under optimal configurations, the sensor demonstrates successful gas-sensing modulation at room temperature. The structural insertion of trivalent Al^3+^ into the divalent A2^3+^ oxide matrix effectively alters carrier concentration and creates surface defects, facilitating low-temperature gas chemisorption and enhancing the film’s electronic response properties.

S. Zhang et al. explored the impact of temperature-driven morphological evolution on the gas-sensing characteristics of p-type NiO nanostructures [93]. Using a sacrificial NiC_2_O_4_ precursor template, the authors synthesized two unique architectures: porous nanorods (PNRs) via annealing at 370 °C and coral-like nanochains (CNCs) via annealing at 450 °C. Gas-sensing performance tests reveal that both sensor variants attained an optimal operating temperature of 320 °C. Despite experiencing a significant reduction in the Brunauer–Emmett–Teller (BET) specific surface area (from 225.05 m^2^/g to 51.06 m^2^/g), the CNC sensor vastly outperforms the PNR configuration. Across a wide concentration range (100–4000 ppm), the CNC sensor generates a response of 55.8% to 4000 ppm of methane, more than doubling the response of the PNR sensor (26.9%). X-ray photoelectron spectroscopy (XPS) analysis manifests that the nanochain morphology accommodates a higher concentration of surface oxygen vacancies (29.4% vs. 27.4%), which promotes heightened charge carrier modulation and vastly accelerates surface chemisorption dynamics.

(2) Cupper Oxide (Cu_2_O, CuO)

Cu_2_O is a p-type semiconductor with bandgap in the range of 1.8–2.5 eV. The Cu_2_O has a high conductivity and Hall mobility at low temperatures. The thermal activation energy (ΔE) of conductivity of Cu_2_O was reported in the 0.2–0.35 eV range depending on the preparation conditions. Jayatissa et al. successfully fabricated high-performance methane gas sensors utilizing p-type cuprous oxide (Cu_2_O) thin films [94]. Synthesized via vacuum evaporation followed by thermal oxidation of copper films, the resulting Cu_2_O layers exhibit a porous structure with a preferred (111) crystal orientation. Characterization reveals a low carrier density (3 × 10^15^ cm^−3^) alongside high conductivity. The sensors demonstrated exceptional reproducibility, fast response times, and an optimal operating temperature of just 180 °C. This efficient performance at low temperatures is attributed to significant grain-boundary depletion layer modulation upon methane exposure, making it a promising candidate for scalable, low-power sensor integration.

Moreover, copper monoxide, CuO, has been investigated because of its strongly correlated electron system and its Cu–O planes in high-temperature superconductors. N. M. Shaalan et al. demonstrate an efficient, low-cost approach to methane gas detection utilizing pure p-type copper oxide (CuO) nanocrystals [95]. Fabricated via a fast microwave-assisted combustion method using copper nitrate and urea, the resulting monoclinic CuO nanocrystals exhibit a spherical morphology with an average crystallite size of ~4–5 nm. Gas-sensing evaluations across temperatures from 150 °C to 300 °C reveal that the sensor achieves its peak response (R_g_/R_a_ = 2.91) to 1% methane at an operating temperature of 300 °C. Resistance increases upon methane exposure, confirming classic p-type behavior. This sensing mechanism is driven by the reaction of methane with chemisorption-induced surface oxygen species (O^−^), which injects electrons back into the CuO core to trigger electron-hole recombination and modulate conductivity.

(3) Cobalt Oxide (Co_3_O_4_)

Co_3_O_4_ possesses a spinel-type structure with two oxidation states of Co^2+^ and Co^3+^, among which the reduction of the latter to the former is handy to enhance the formation of oxygen vacancies at low temperatures. Assisted by oxygen vacancies, high oxygen mobility and highly active surface oxygen species facilitate the activation and breaking of C–H bonds, leading to reduced energy for methane dissociation via direct interaction of C–H orbitals with d-type orbitals of Co cations.

P. Chesler et al. investigated the potential of cobalt oxide (CoO)-based chemiresistors for low-concentration methane detection as cost-effective alternatives for methane safety monitoring [96]. CoO thin films were synthesized via an eco-friendly sol–gel deposition method and integrated onto alumina substrates equipped with Au or Pt interdigitated electrodes. Atomic force microscopy (AFM) and scanning electron microscopy (SEM) analyses revealed highly rough, porous film morphologies that promote methane adsorption and sensing performance. Gas-sensing evaluations across 5–2000 ppm CH_4_ demonstrated that CoO-based sensors achieved high sensitivity, rapid response/recovery (~250 s), and stable operation at relatively low working temperatures (210–220 °C). Humidity and CO_2_ interference tests confirmed partial selectivity, with the CoO-based sensor exhibiting superior methane discrimination and overall 3-S performance (sensitivity, selectivity, and stability).

G. T. Smagulova et al. introduced a novel PANI/Co_3_O_4_ core–shell nanocomposite designed for room-temperature chemiresistive detection of methane to improve fire-safety monitoring [97]. Cobalt oxide nanoparticles were fabricated using solution combustion synthesis (SCS), yielding a porous, defect-rich Co_3_O_4_ spinel matrix. This matrix was encapsulated by a conducting polyaniline (emeraldine salt) shell via low-temperature controlled oxidative polymerization. Characterized by a highly porous core–shell architecture, the composite sensor demonstrates effective room-temperature gas diffusion and reversible electronic signaling under ambient conditions without external heating. Upon exposure to 500 ppm of target gases, the sensor yields distinct resistance decreases, achieving initial responses of 8.73% for methane. This performance enhancement is attributed to the synergistic interplay between the reversible Co^3+^/Co^2+^ redox couple on the Co_3_O_4_ core, modulation of the polyaniline protonation equilibrium, and efficient polaron-mediated charge transport through the polymer network.

P-type copper-doped cobalt oxide (Cu/Co_3_O_4_) nanoparticles were developed by Z. S. Mehrabadi et al., who studied their structural optimization and methane gas-sensing [98]. Synthesized via a low-temperature sol–gel method with varying Cu/Co mole ratios (0.05 to 0.15), XRD and TEM analyses suggest the stable formation of cubic Co_3_O_4_ mixed with a secondary (CuO_0.3_CoO_0.7_)Co_2_O_4_ spinel phase. Copper incorporation systematically degraded overall crystallinity, effectively reducing the average crystallite size from 28 nm down to 24 nm. Via sensing experiments conducted between 200 °C and 300 °C, 300 °C is identified as the optimal operating temperature across a sub-explosive methane concentration spectrum (3000–6000 ppm). The sensor performance scales positively with increased copper loading, reaching its peak sensitivity at a Cu/Co mole ratio of 0.15. This structural improvement is explicitly ascribed to enhanced carrier modulation over the smaller, high-surface-area nanocrystals and the added catalytic influence of copper on surface chemisorption pathways.

### 3.3. Quantitative Comparison of Representative CH_4_ Sensors

It is important to clarify that the gas response values reported for CH_4_ sensors in the literature are not defined uniformly. In the studies discussed in this review, the response can be expressed in several ways. For n-type materials such as In_2_O_3_, SnO_2_, and ZnO, a common definition is the resistance ratio S = R_a_/R_g_, where R_a_ is the baseline resistance in air, and R_g_ is the resistance in the presence of the target gas. For p-type materials like NiO and CuO, the response is often defined as S = R_g_/R_a_. Many authors, however, report percentage response as S (%) = (R_a_/R_g_) × 100% for n-type or S (%) = (ΔR/R_a_) × 100% for both types. In a few cases, the response is expressed simply as the relative resistance change. Therefore, the response magnitudes listed in Table 3 are reproduced as they appear in the original cited works and are not directly comparable without referring to the specific definition and test conditions used in each study. This caveat applies equally to the CO sensor data summarized in Table 4.

Table 3 summarizes representative CH*_4_* sensors discussed in this review. The values are reproduced from the cited studies as reported in this manuscript; response definitions differ among papers (e.g., Rg/Ra, Ra/Rg, percentage change, or other formulations), so numerical response values should not be ranked without considering the definition, concentration, and test conditions. The table therefore emphasizes the combined performance envelope.

## 4. MO Carbon Monoxide Gas Sensors

### 4.1. n-Type MO

(1) Indium oxide (In_2_O_3_)

S. B. Patil et al. present a highly efficient, n-type chemiresistive methane gas sensor featuring nanocrystalline indium oxide thin films synthesized via a low-cost sol–gel dip-coating method [99]. Comprehensive structural characterization (XRD, FESEM, AFM, TEM, and SAED) confirms the formation of a pure cubic phase consisting of homogeneously dispersed, spherical nanoparticles with an average crystallite size of 13 nm. As evaluated for carbon monoxide (CO) detection, the sensor achieved an exceptional maximum response (S = 589) to 50 ppm CO under optimal operating parameters of 350 °C and 10 V. Notably, the nanostructured geometry facilitates rapid gas transport, yielding an ultra-fast response time of 7 s and a recovery time of 8 s. Furthermore, the thin-film sensor demonstrates excellent long-term stability and outstanding selectivity toward CO over other interfering gases like LPG, NH_3_, and H_2_S.

Y. Sun et al. clarified the gas-sensing mechanism of n-type indium oxide nanoparticles synthesized via a precipitation method for carbon monoxide (CO) detection [100]. By evaluating the mathematical relationship between electrical resistance and oxygen partial pressure (P_O2_), the researchers demonstrate that resistance scales linearly with P_O2_^1/4^ across both dry and wet conditions. This directly proves that O^2−^ serves as the primary adsorbed oxygen species on In_2_O_3_, distinguishing its behavior from SnO_2_. Using a competitive adsorption model, the adsorption equilibrium constants (K_1_ for O^−^ and K_2_ for O^2−^) were calculated. Both constants reached a maximum value at 300 °C, aligning perfectly with the sensor peak operating response to 8 ppm CO. While humidity compromises overall performance through hydroxyl poisoning, the K_2_/K_1_ ratio exhibits a strong linear correlation with sensor sensitivity. The findings confirm that O^2−^ chemisorption dictates the sensor’s overall responsiveness and plays a more vital role than O^−^ in driving target gas interactions.

D. Zhang et al. report a high-performance, room-temperature carbon monoxide (CO) gas sensor using a cobalt-doped indium oxide (Co-In_2_O_3_) nanoparticle/molybdenum disulfide (MoS_2_) nanoflower ternary nanocomposite [101]. Synthesized via co-precipitation and hydrothermal methods, the film was successfully fabricated using a layer-by-layer self-assembly technique. Material characterization confirmed excellent structural integrity and morphology. Gas-sensing experiments demonstrated outstanding repeatability, long-term stability, rapid response/recovery kinetics, and superior sensitivity compared to pristine controls. Density-functional theory (DFT) simulations revealed that the underlying sensing mechanism is heavily driven by enhanced oxygen vacancy generation from Co^2+^ doping and electronic modulation at the Co-In_2_O_3_/MoS_2_ n-n heterojunction interfaces.

**Figure 8:**
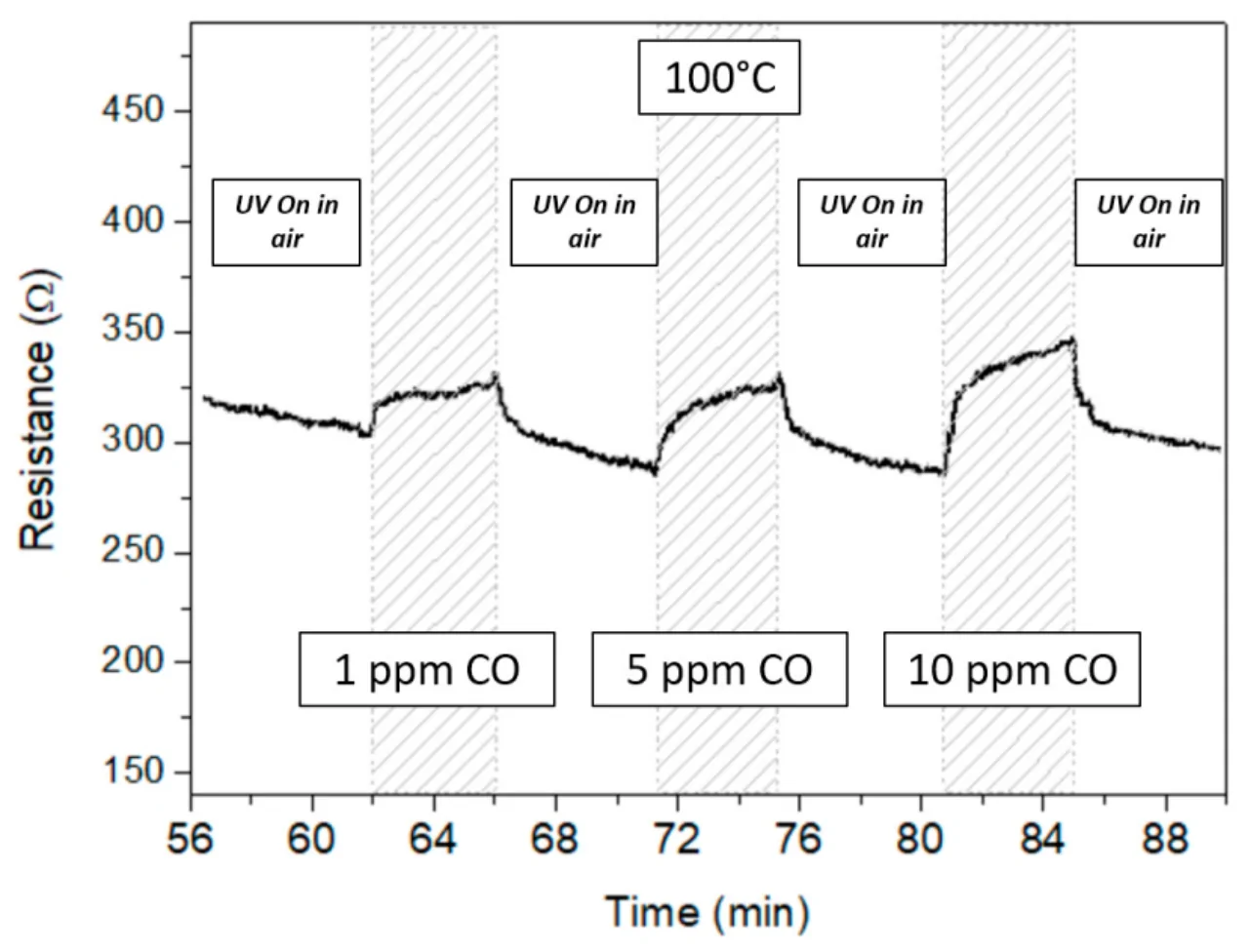
Transient sensor response at T = 100 °C and different CO concentrations in “UV On in air” mode. Reproduced from Reference [102], with permission from CC-BY-NC 4.0.

A. Malara et al. synthesized In_2_O_3_ powder via precipitation from indium nitrate solution with potassium carbonate, followed by calcination at 500 °C (Figure 8) [102]. XRD confirmed a highly crystalline cubic bixbyite phase with crystallite size ~30 nm, while SEM showed cubic particles assembled in agglomerates (0.1–0.8 μm). The BET surface area was 20.3 m^2^/g with a pore volume of 87 mm^3^/g. Gas-sensing tests revealed that continuous UV irradiation did not improve CO response. However, when the UV LED irradiated the sensor only during regeneration in airflow (“UV On in air” mode) and was turned off during CO detection, an apparent p-type behavior emerged, yielding higher responses at 100 °C toward 1–10 ppm CO. Under this optimized condition, the sensor achieved a limit of detection below 200 ppb, demonstrating practical potential for sub-ppm CO monitoring at low temperatures.

(2) Tin oxide (SnO_2_)

A. Kolmakov et al. reported the fabrication and evaluation of highly uniform tin oxide nanowires for high-performance carbon monoxide and oxygen gas-sensing applications [103]. Synthesized via the isolation and subsequent oxidation of template-grown tin nanowires, the resulting nanostructures demonstrate excellent stability, fast response/recovery kinetics, and strong reproducibility. The underlying chemiresistive sensing mechanism operates on the principle that the nanowires' bulk electronic properties are directly governed by their surface chemistry. Exposure to target gases triggers molecule adsorption/desorption processes that modulate electron trapping and surface conductivity, offering an efficient strategy for integration into low-power, multi-component sensor arrays. Moreover, V. Ambardekar et al. evaluate the impact of morphology on CO sensing performance of tin dioxide elements by comparing an atmospheric plasma-sprayed thick film coating against its bulk counterpart [104]. The thick film, featuring a smaller average particle size (52.95 nm) and a combination of mesopores and macropores, exhibited superior gas responses and baseline resistances due to enhanced oxygen adsorption.

V. Kishnani et al. present a high-performance carbon monoxide (CO) gas sensor operating at ambient temperature using a ternary SnO_2_/PANI/Pd nanocomposite film [105]. The hybrid material was synthesized via a hydrothermal route, successfully integrating n-type tin oxide nanoparticles, conducting polyaniline (PANI), and catalytic palladium (Pd) clusters. Gas-sensing characterization reveals that the SnO_2_/PANI/Pd composite exhibits exceptional sensitivity, achieving a maximum response (R_a_/R_g_) of 4.19 toward 100 ppm CO at room temperature. The sensor demonstrates rapid response (52 s) and recovery (41 s) kinetics, excellent long-term stability, and prominent selectivity against interfering gases like carbon dioxide and methane. This synergistic performance enhancement is driven by the catalyst-assisted spill-over effect of Pd, high surface area for gas adsorption, and multi-interface charge modulation across the p-n heterojunctions formed between PANI and SnO_2_.

H. Long et al. present a facile method for high-performance carbon monoxide (CO) gas sensors by generating porous tin oxide thin films directly in situ on low-power microheater platforms [106]. Using the microheater itself to rapidly anneal liquid metal–organic precursors locally, a nanostructured, highly crystalline, and porous sensing layer is formed with minimal processing. When evaluated for CO detection, the integrated microheater configuration enables sensitivity and rapid response/recovery kinetics at significantly reduced operating temperatures. This localized synthesis technique bypasses traditional high-temperature furnace steps, offering an efficient, scalable approach for manufacturing miniaturized, low-power environmental monitoring arrays.

(3) Zinc oxide (ZnO)

F. Schipani et al. detailed the fabrication of zinc oxide thin films via spray-pyrolysis for room-temperature gas-sensing applications [107]. The thickest film exhibits the highest sensitivity, a feature directly credited to the dominant exposure of the reactive (100) crystallographic facet. Operating successfully without integrated microheaters, the sensor presents a pronounced sensitivity to carbon monoxide at occupational safety thresholds (50 ppm), which allows distinct signal differentiation, rendering it an effective, low-power CO environmental monitor.

L. Utari et al. fabricated a flexible wearable chemiresistive CO sensor based on graphene/ZnO nanocomposites grown on cotton fabrics [108]. The sensor was constructed by depositing graphene onto textile substrates through dip-coating, followed by the growth of vertically aligned ZnO nanorods via chemical bath deposition. Structural and morphological analyses confirmed the formation of hexagonal wurtzite ZnO nanorods with diameters of approximately 30 nm and lengths of 1.5 μm on the graphene layer. Operating at room temperature (27 °C), the optimized graphene/ZnO sensor exhibited enhanced CO sensing performance, detecting concentrations down to 10 ppm with response and recovery times of 280–435 s and 45–115 s, respectively. The sensor achieved a 40.26% response to 50 ppm CO with selectivity over NO, ethanol, acetone, and methanol. The improved performance was attributed to the graphene/ZnO heterojunction, which facilitated electron transfer and enhanced oxygen-mediated CO oxidation reactions at the sensing interface.

**Figure 9:**
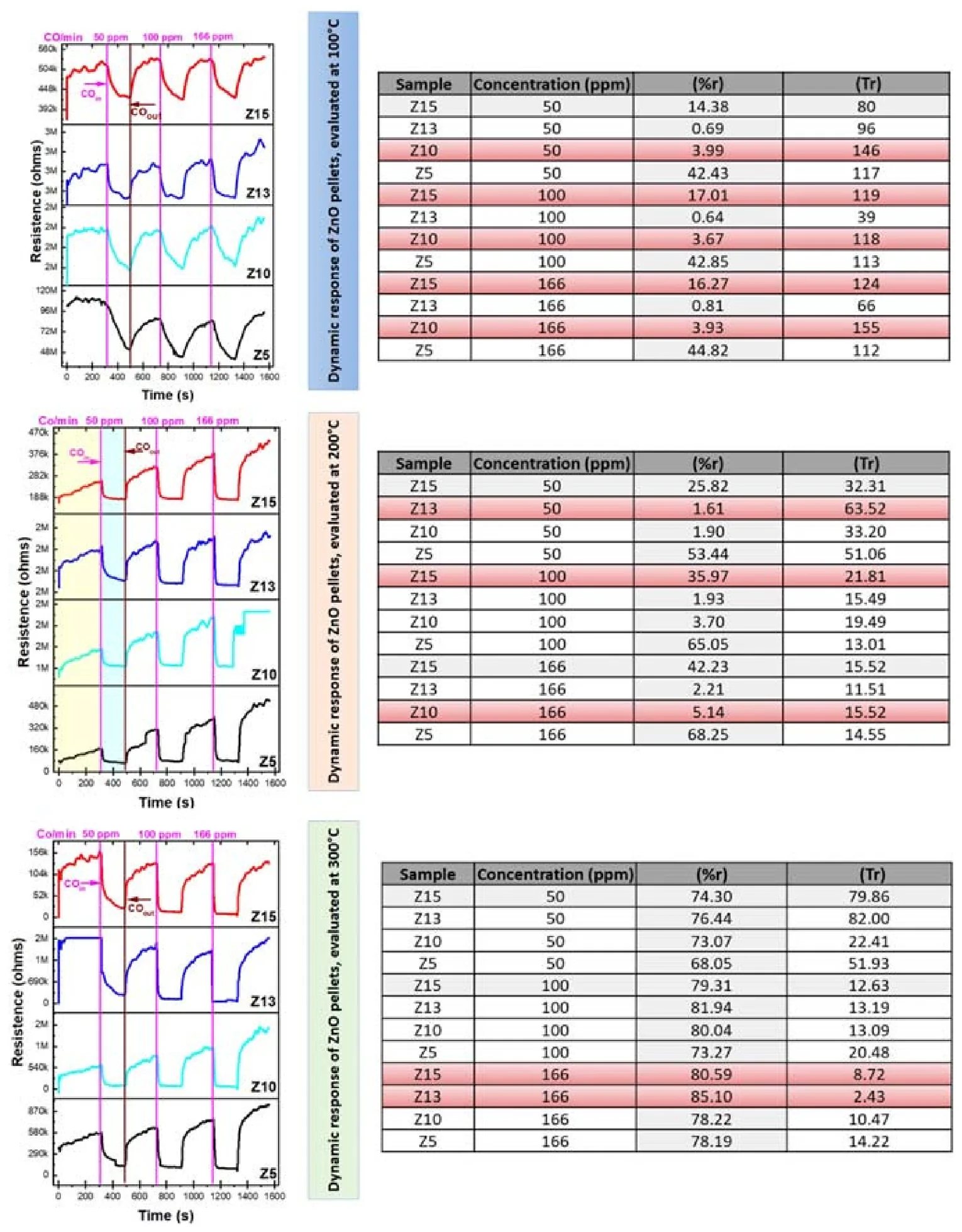
Dynamic response diagrams and tables of Z5, Z10, Z13, and Z15samples at 50, 100, and 166 ppm of CO. Reproduced from Reference [109], with permission from CC-BY-NC 4.0.

As illustrated in Figure 9, M. P. Munguia-Martin et al. synthesized ZnO structures via the chemical precipitation method using urea as precipitating agent, followed by thermal treatment at 500 °C for 5, 10, 13, and 15 h [109]. XRD confirmed a hexagonal wurtzite phase with crystallite sizes ranging from 14.68 to 18.95 nm. SEM revealed spherical and semi-spherical agglomerates, with particle size decreasing from 445.90 nm (Z5) to 143.43 nm (Z13) as treatment time increased, attributed to enhanced dispersion and fragmentation; a further increase to 15 h caused partial particle fusion and densification. Gas-sensing tests conducted on CO (50–166 ppm) at 100–300 °C demonstrated that the Z13 sample exhibited the fastest response time (2.43 s at 166 ppm, 300 °C) and the highest sensing response among all samples due to its smallest particle size providing larger surface area and more active sites for oxygen adsorption and CO oxidation.

Zhang et al. successfully fabricated a novel ternary Ag-ZnO/MoS_2_ nanocomposite film for room-temperature carbon monoxide gas sensing using a layer-by-layer self-assembly technique [110]. Comprehensive structural characterizations reflect the synergistic integration of metallic Ag nanoparticles, ZnO nanorods, and layered MoS_2_ nanosheets. The sensor exhibits outstanding room-temperature sensing performance under exposure to CO concentrations ranging from 1 to 1500 ppm, yielding high sensitivity, swift response/recovery kinetics (45–60 s/40–50 s), acceptance-level repeatability, long-term stability, and selectivity against competing gases. This performance significantly outstrips pristine ZnO, binary ZnO/MoS_2_, and Pt-ZnO/MoS_2_ alternatives. The superior performance is convincingly credited to the catalytic behavior of Ag, the material synergy between ZnO and MoS_2_, and a lowered Schottky barrier height governed by the low work function of silver.

(4) Tungsten Oxide (WO_3_)

M. Hossain et al. explore the unconventional carbon monoxide (CO) gas-sensing behavior of oxygen-deficient, n-type phase-separated tungsten oxide WO_3−x_ 2D nanosheets [111]. Unlike pristine WO_3_, which demonstrates a conventional resistance decrease under reducing CO (−84% at 10 ppm), the WO_3-x_ matrix—comprising poorly insulating WO_2.9_ domains embedded within a highly insulating WO_2_ host—displays an inverted, anomalous resistance increase (94% at 10 ppm). Backed by density functional theory (DFT) calculations, this atypical CO response is governed by site-specific chemisorption onto distinct competing surfaces of the spatially separated 2D oxide phases, unlocking unique cross-sensitivity mechanisms for mixed-gas ambient monitoring.

M. A. Syaahiran et al. conducted a density functional theory (DFT) study on carbon monoxide (CO) adsorption on pristine graphene, tungsten oxide/graphene (g-W_n_O_3n_), and chromium-doped tungsten oxide/graphene (g-CrW_n−1_O_3n_) composites for gas sensor applications [112]. Calculated at the B3LYP/LanL2DZ level, the results reveal that Cr-doping significantly reduces the HOMO-LUMO energy gap and enhances surface reactivity. The CO interaction demonstrates an exothermic physisorption mechanism supported by substantial charge transfer, electrostatic potentials, and distinct infrared and photoelectron spectroscopy shifts. Notably, the g-CrWO_6_ (*n* = 2) composite exhibits outstanding sensitivity, swift charge polarization, and balanced desorption recovery, establishing it as a highly promising candidate for advanced CO detection.

Marikutsa et al. examine the effects of microstructure and surface modification (1 wt.% Pd or Ru) on nanocrystalline monoclinic γ-WO_3_ for resistive gas sensing [113]. Through temperature-programmed techniques and dynamic tests, the authors demonstrate that increasing particle size reduces surface acidity and hydroxyl populations. Crucially, in situ DRIFT spectroscopy reveals that the catalytic additives modify the gas–solid interaction pathways. Palladium modification optimizes room-temperature CO detection by enabling chemisorption and subsequent oxidation via surface aqueous species. Conversely, ruthenium modification suppresses the oxygen-vacancy reduction route inherent to pristine WO_3_, instead facilitating high-temperature NH_3_ sensing through Ru-catalyzed oxidation into nitrosyl species. This underscores a uniform chemical modification model applicable across diverse semiconductor metal oxides.

(5) Vanadium Oxide (V_2_O_5_)

Yepuri et al. present a straightforward, dopant-free strategy to fabricate crystalline vanadium oxide thin films (~25 nm) via sol–gel spin-coating, optimized through air-annealing at 500 °C [114]. Comprehensive material characterization (XRD, FTIR, FESEM, and EDAX) confirms a dense, chemically pure, nanogranular morphology. UV–Vis spectroscopy reveals an optical bandgap of 2.67 eV. Crucially, the authors establish a direct structure–property–performance correlation, showing that annealing-induced microstructural changes govern charge transport. The sensor demonstrates stable, reproducible carbon monoxide (CO) detection across 10–500 ppm, yielding a maximum resistance modulation of ~25.5 M\Omega at a remarkably low operating temperature of 100 °C. This work offers an efficient pathway for low-power gas-sensing configurations.

Ashok et al. detail the synthesis of mixed-phase titanium–vanadium oxide (TVO) thin films via thermal oxidation of evaporated metal layers [115]. Multi-technique characterization (XRD, Raman) confirms the co-existence of orthorhombic V_2_O_5_, tetragonal TiO_2_, and monoclinic V_2_Ti_3_O_9_ phases, with crystallinity and grain size (50–200 nm) increasing alongside post-annealing temperature (500–575 °C). The indirect bandgap spans 2.14–2.36 eV, and negative Hall coefficients establish n-type conductivity. For CO gas sensing (10–500 ppm), surface electrical resistance variations prove that the TVO film optimized at 550 °C achieves peak response and sensitivity at a low operating temperature of 100 °C.

### 4.2. p-Type MO

(1) Nickel Oxide (NiO)

M. Li et al. investigated the CO sensing mechanism of nickel oxide through operando diffuse reflectance infrared Fourier transform spectroscopy (DRIFTS) combined with resistance measurements [116]. The NiO sensor exhibited stable responses toward CO and H_2_, with limited influence from humidity, demonstrating the robustness of NiO under realistic operating conditions. The study revealed that CO sensing was governed by oxidation–reduction processes occurring at the NiO surface rather than the formation of highly specific adsorbed CO intermediates. CO molecules reacted with surface oxygen species, resulting in partial reduction of NiO and modification of the hole concentration, which produced measurable resistance changes. The authors emphasized that NiO behaves differently from many conventional n-type metal oxides due to its p-type conduction mechanism and relatively low humidity sensitivity. These findings provided important insights into the fundamental sensing chemistry of NiO-based CO chemiresistors.

Khaleed et al. investigated the enhancement of carbon monoxide sensing by incorporating activated carbon (AC) into nickel oxide [117]. Flower-like NiO/AC composites were successfully synthesized via a hydrothermal reflux process and calcined at 400 °C. Characterization via XRD, SEM, and HRTEM indicates that grain growth is restricted by adding amorphous AC, yielding a high specific surface area of 59.91 m^2^g^−1^. At an operating temperature of 100 °C, the p-type chemiresistive composite sensor shows significantly improved electrical conductivity, higher response percentages (up to 100 ppm CO), and accelerated response/recovery durations as compared to pristine NiO.

Nagarajan and Chandiramouli theoretically investigated the carbon monoxide (CO) adsorption properties of a p-type NiO nanocone using density functional theory (DFT) with the B3LYP/LanL2DZ basis set [118]. The computational results reveal that the most energetically favorable adsorption site for CO is the oxygen atom situated at the apex of the NiO nanocone. This configuration achieves a highly exothermic adsorption energy of −8.432 eV and triggers a significant 50% variation in the average energy gap. Mulliken population and density of states (DOS) analyses confirmed effective electron transfer from CO to the oxygen site, which decreases the majority of carrier holes and broadens the energy gap. Consequently, this structural interaction drastically modulates electrical conductivity, suggesting that synthesizing oxygen-rich NiO in a nanocone morphology can profoundly optimize and enhance its sensitivity for CO gas-sensing applications without requiring additional chemical functionalization.

**Figure 10:**
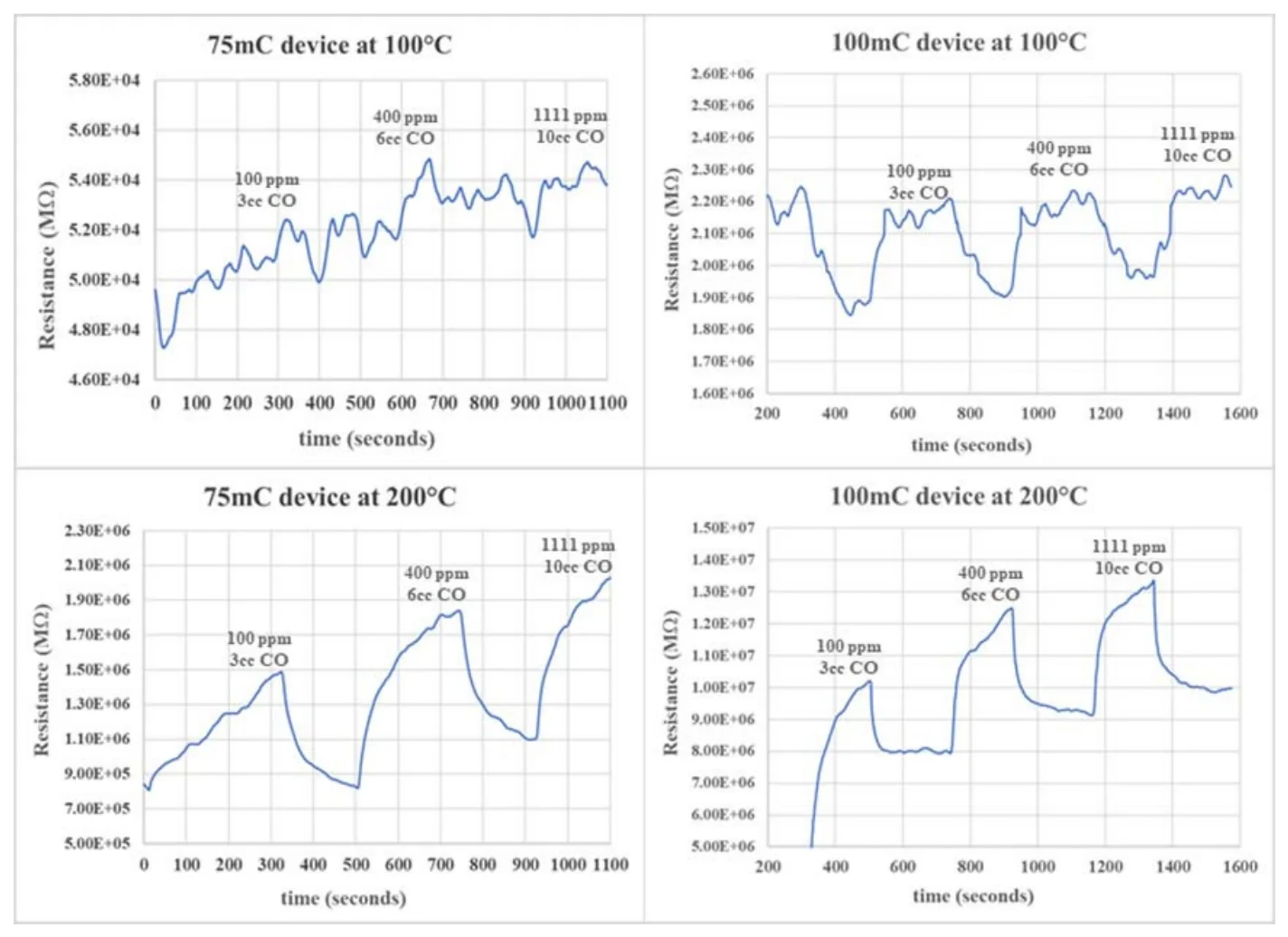
Dynamical response of NiO devices towards 100, 400, and 1111 ppm of CO at 100 and 200 °C. Reproduced from Reference [119], with permission from CC-BY-NC 4.0.

As shown in Figure 10, A. S. Ivan et al. synthesized NiO thin films via the sol–gel method combined with deep coatings, using nickel acetate tetrahydrate and methanol as precursors, followed by thermal treatment at 500 °C for 1 h [119]. XRD confirmed a pure face-centered cubic NiO phase, while SEM revealed homogeneous nodular nanoparticles (10–15 nm) with 75 nm thickness. TEM showed irregular polycrystalline particles (~20 nm), and UV–Vis analysis yielded a band gap of 3.3 eV, consistent with p-type semiconductors. Static environment tests at 100–300 °C toward CO (1–300 ppm) showed that the 75 μm-spacing device exhibited excellent sensitivity at low concentrations (1 ppm) with 51% at 50 ppm and 300 °C. Flow-through measurements demonstrated the 100 μm spacing device performed optimally at 200 °C for 100 ppm CO, with response and recovery times of 165 s and 212 s, respectively, making it suitable for aircraft cabin CO detection.

(2) Copper Oxide (Cu_2_O, CuO)

**Figure 11:**
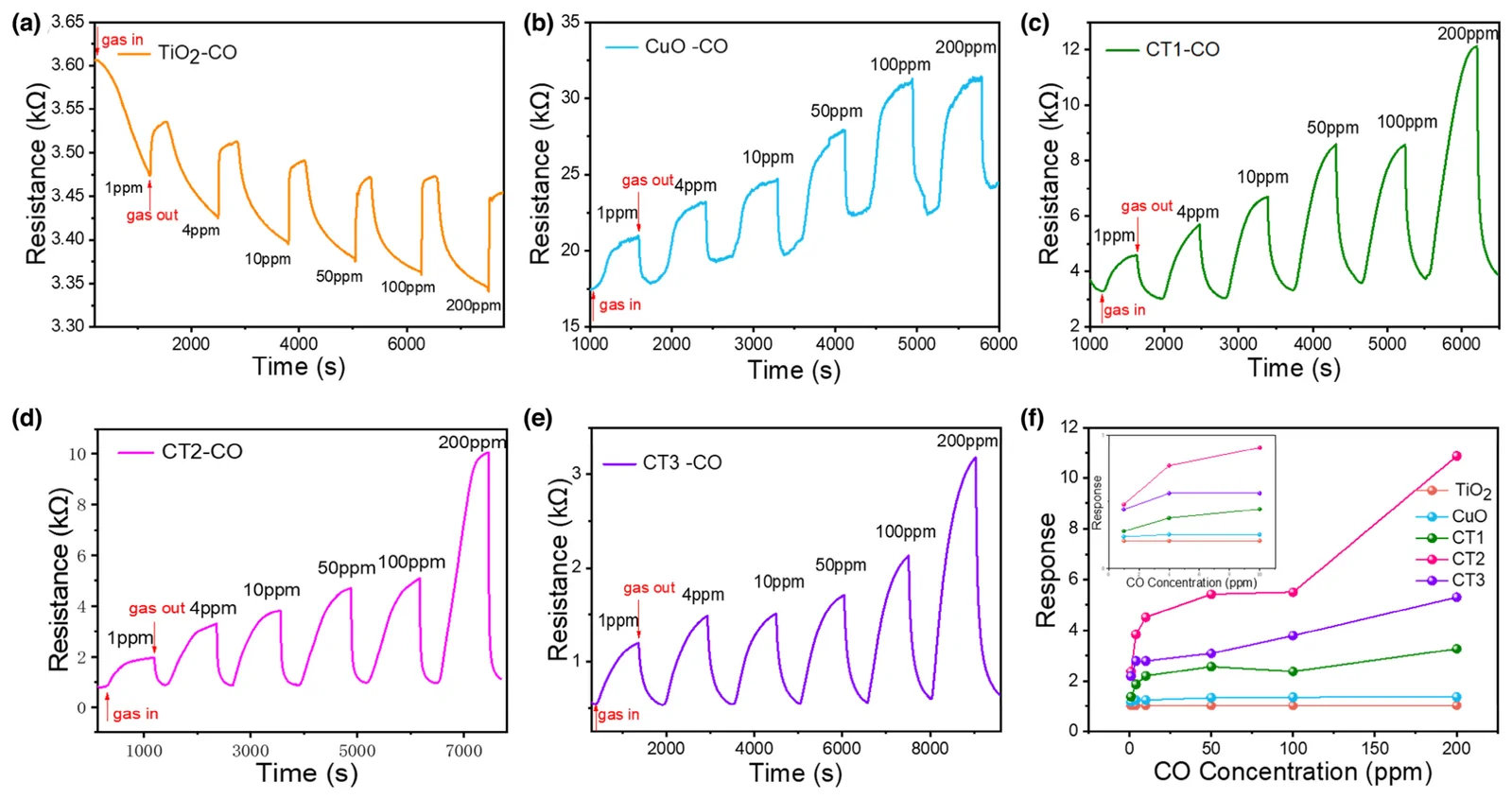
Carbon monoxide sensing curves: resistance change as a function of time at different CO concentrations measured at a room temperature of 25 °C: (**a**) TiO_2_, (**b**) CuO, (**c**) CT1, (**d**) CT2, and (**e**) CT3. (**f**) The comparison of the response of TiO2, CuO, CT1, CT2, and CT3. Reproduced from Reference [120], with permission from CC-BY-NC 4.0.

As displayed in Figure 11, W. Wei et al. successfully fabricated CuO/TiO_2_ heterojunction thin-film sensors via a two-step hydrothermal method, with self-assembled [002]-oriented TiO_2_ nanorods as the bottom n-type layer and CuO nanosheets with (111) exposed facets as the top p-type layer [120]. XRD confirmed the coexistence of rutile TiO_2_ and monoclinic CuO phases, while SEM and TEM revealed intimate interfacial contact between TiO_2_ nanorods and CuO nanosheets, significantly increasing the heterojunction area. XPS analysis indicated abundant oxygen vacancies and adsorbed oxygen species on the heterojunction surface. Gas-sensing tests at room temperature (25 °C) demonstrated that the optimized CT2 sensor exhibited a high response of 10.8 to 200 ppm CO, which was about 10 times its response to H_2_, achieving an interference factor IF (CO, H_2_) of 8.9. The enhanced selectivity is attributed to the good CO adsorption properties of CuO and the heterojunction interface charge transfer that suppresses H_2_ interference. L. Hou et al. investigated the influence of morphology and crystal facet orientation on the CO gas-sensing capabilities of p-type CuO nanomaterials [121]. The authors successfully synthesized CuO nanotubes (NTs) exposing the (111) facet and CuO nanocubes (NCs) exposing the (110) facet via controlled thermal oxidation. Characterization shows that the hollow, highly porous CuO NTs possess a significantly larger specific surface area (143.2 m^2^/g) compared to the solid CuO NCs (82.4 m^2^/g). Gas-sensing evaluations revealed that CuO NTs outperform the NCs, demonstrating a lower optimal operating temperature (175 °C vs. 250 °C), superior sensitivity (2.73 × 10^−3^ ppm^−1^), enhanced selectivity, and a lower limit of detection (0.6 ppm). While the NTs exhibited longer response and recovery times due to their porous framework, both sensors operated in under one minute. Ultimately, this research underscores how targeted surface crystal engineering can dramatically optimize metal-oxide semiconductor gas sensors.

Steinhauer et al. present a novel, cost-effective method for fabricating conductometric gas sensors based on CuO nanowire bridges [122]. Utilizing a low-temperature (400 °C) thermal oxidation process on electroplated copper microstructures, the authors successfully integrated suspended polycrystalline CuO nanowires directly on-chip. This backend-compatible CMOS approach avoids complex post-synthesis assembly. Thanks to an optimized surface-to-volume ratio, the devices achieved excellent detection limits for carbon monoxide (10 ppm) and hydrogen sulfide (10 ppb). Interestingly, humidity decreased CO sensitivity but enhanced H_2_S response, making it a promising candidate for smart silicon-integrated safety applications.

Oosthuizen et al. successfully synthesized pure monoclinic p-type CuO nanoplatelets via a surfactant-free hydrothermal method to monitor indoor air quality [123]. Structural, magnetic, and electrical evaluations showed that the CuO-B-1 sample—prepared at 200 °C for 6 h—exhibits optimal crystallinity, minimized grain boundaries, low resistivity, and high charge carrier mobility. Consequently, the CuO-B-1 sensor achieved exceptional room-temperature cross-selectivity and a response value of 15.1 toward 20 ppm CO under 35% relative humidity. This robust performance, combined with stable 8-day repeatability, establishes it as a highly promising candidate for practical environmental safety applications.

(3) Cobalt Oxide (Co_3_O_4_)

T. D. Nguyen et al. report a novel aqueous hydrothermal strategy to synthesize mesoporous CoO/Co_3_O_4_/WO_3_ heterostructured nanotoroids, utilizing aminocaproic acid as a key structure-directing agent [124]. This amino acid-mediated self-aggregation successfully yields unique hierarchical, ring-like mixed-metal oxide complexes featuring distinct nanoscale porosity. Exploiting the structural synergy of a macro-porous toroidal geometry, high intrinsic porosity (70 m^2^/g), and a well-defined p-n semiconductor heterojunction, the authors fabricated highly sensitive chemiresistive gas sensors. The resulting platforms demonstrated outstanding, stable, and reversible electrical response characteristics alongside rapid diffusion/recovery kinetics (20–40 s) toward reductive analytes like CO at an optimized operating temperature of 400 °C.

**Figure 12:**
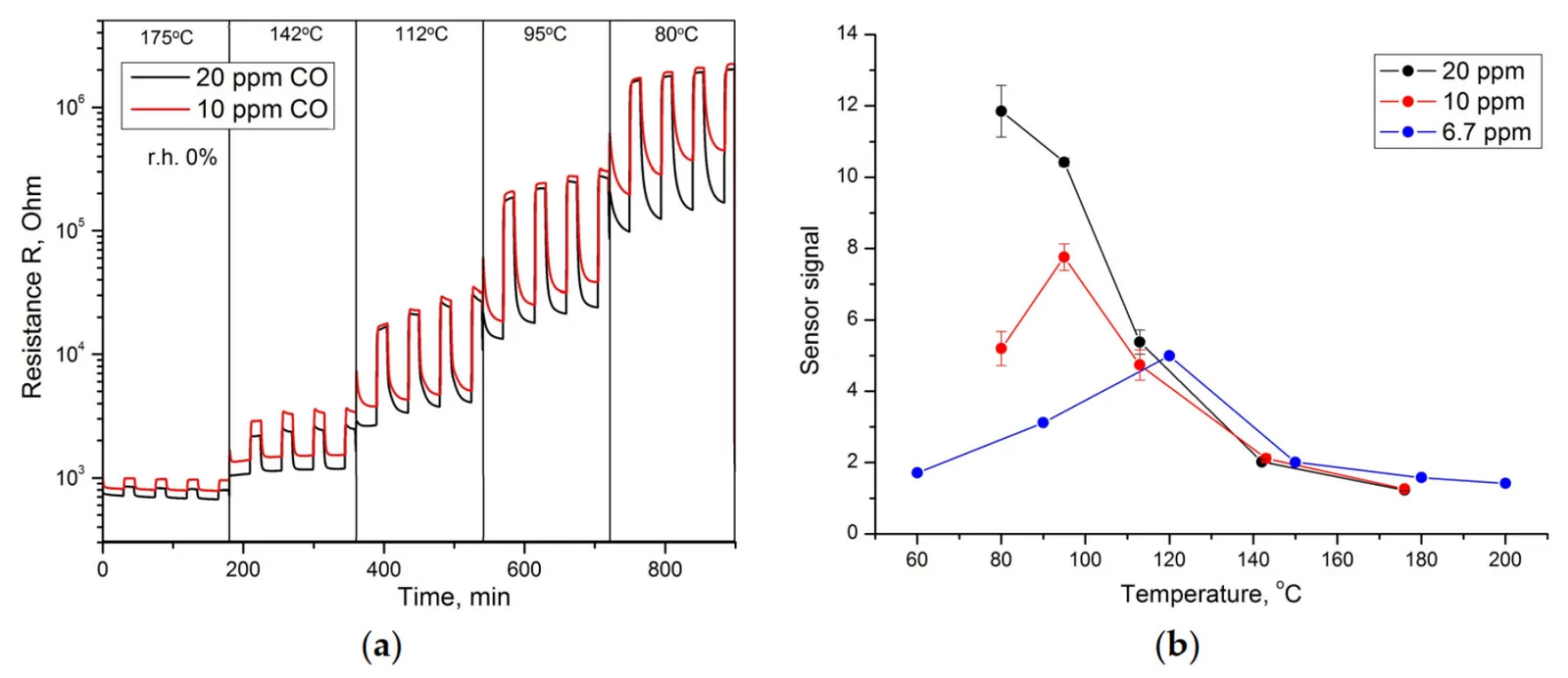
(**a**) Electrical response of Co_3_O_4_ to the periodic change in gas phase composition from dry air (30 min) to CO/air (15 min); (**b**) sensor response of the Co_3_O_4_ to CO (0% r.h.) as a function of temperature. Reproduced from Reference [125], with permission from CC-BY-NC 4.0.

As shown in Figure 12, S. Vladimirova et al. investigated nanocrystalline Co_3_O_4_ as a p-type chemiresistive sensing material for carbon monoxide detection under both dry and humid conditions [125]. Co_3_O_4_ nanoparticles were synthesized through precipitation, followed by thermal decomposition of a carbonate precursor and characterized using XRD, TEM, and FTIR techniques. The sensor demonstrated CO responses in the concentration range of 6.7–20 ppm, with effective sensing behavior at moderate temperatures. The influence of humidity was systematically studied, revealing that water vapor significantly affected the CO response at low temperatures (80–120 °C), while stable sensing performance was achieved between 180 and 200 °C under different humidity levels. The sensing mechanism was associated with CO oxidation involving chemisorbed oxygen species and the reversible modulation of hole concentration in p-type Co_3_O_4_. The study highlighted Co_3_O_4_ as a promising oxide material for CO detection in realistic environments.

M. Alheshibri et al. investigated an environmentally friendly strategy for synthesizing nanostructured spinel cobalt oxide sensors using pulsed laser ablation in liquid (PLAL). By regulating the ethanol/water concentration in the ablation media, the authors achieved precise control over the material morphology, which transitions from solid/hollow spheres to ultra-thin nanosheets/flakes. Comprehensive structural analyses (TEM, XRD, XPS, and Raman spectroscopy) reveal that the Co_3_O_4_ nanosheets prepared at 70% ethanol possess enriched oxygen vacancies and a high surface area. Consequently, this morphology displayed superior p-type chemiresistive sensing performance, delivering a maximum sensitivity of 360% toward 200 ppm of carbon monoxide gas at an operating temperature of 300 °C [126].

S. Hussain et al. report the successful synthesis of a hierarchical, yeast-like Mo-Co_3_O_4_ nanostructure derived from a cobalt-based metal–organic framework (Co-MOF) precursor via a solvothermal route followed by calcination [127]. The authors thoroughly investigated the material's structural, chemical, and morphological properties, confirming successful Mo^6+^ doping into the Co_3_O_4_ lattice alongside an increase in oxygen vacancies. When evaluated as a chemiresistive sensor for toxic carbon monoxide, the optimum 2 mol% Mo-Co_3_O_4_ formulation demonstrated an outstanding response value of 136 at 100 ppm at a relatively low operating temperature of 200 °C. This performance marks a remarkable 50.4-fold improvement over pristine Co_3_O_4_. Furthermore, the sensor displayed rapid response/recovery kinetics (78.5 s/55.3 s), excellent selectivity against common interfering gases, strong reproducibility, and reliable anti-humidity properties. This work highlights a highly effective morphology-engineering and electronic-tuning strategy for high-performance environmental gas detection.

### 4.3. Quantitative Comparison of Representative CO Sensors

Table 4 applies the same reader-oriented comparison to CO sensors. The selected studies illustrate why low detection limit, high response, low operating temperatures, fast dynamics, humidity tolerance, and selectivity must be considered together.

**Table 4:** Comparisons of Representative CO Sensors.

Material/Architecture	CO Test Concentration	Operating T	Reported Response (and Definition)	LOD	Response/Recovery	Practical Observation	Reference
Nanocrystalline In_2_O_3_ thin film	50 ppm	350 °C	S = R_a_/R_g_ = 589	---	7/8 s	High response and fast kinetics; reported selectivity	[99]
Au/In_2_O_3_	10 ppm	50 °C	S = R_a_/R_g_ = 5.59	---	---	Low-temperature noble-metal modification	[102]
SnO_2_/PANI/Pd	100 ppm	Room T	S = R_a_/R_g_ = 4.19	---	52/41 s	Ambient operation; methane and CO_2_ interference tests reported	[105]
ZnO nanoparticles	80 ppm	250 °C	S (%) = 74% (R_a_/R_g_)	---	21/70 s	One-month stability reported	[109]
Ag-ZnO/MoS_2_	1–1500 ppm	Room T	S = R_a_/R_g_ (high sensitivity)	---	45–60/40–50 s	Broad range; selectivity tests reported	[110]
WO_3−x_ 2D nanosheets	10 ppm	---	S (%) = 94% (resistance increase)	---	---	Unconventional response; phase-separated oxide	[111]
V_2_O_5_ thin film	10–500 ppm	100 °C	Resistance modulation (~25.5 MΩ)	---	---	Low-temperature detection	[114]
NiSb_2_O_6_	1–300 ppm	300 °C	S = R_a_/R_g_ (~0.35 at 300 ppm)	---	---	Alarm circuit demonstrated at 50 ppm	[116]
NiO/activated carbon	up to 100 ppm	100 °C	S (%) = improved vs. NiO	---	Faster than pristine NiO	Composite lowers operating temperature	[117]
NiO/Co_3_O_4_ heterocomposite	CO	150 °C	S (%) = ~150%	---	---	p-p heterojunction and porous architecture	[119]
CuO nanotubes	50–1000 ppm	175 °C	S = 2.73 × 10^−3^ ppm^−1^	0.6 ppm	<1 min	Better than CuO nanocubes; morphology/facet effect	[121]
Mo-doped Co_3_O_4_	100 ppm	200 °C	S = R_g_/R_a_ = 136	---	78.5/55.3 s	50.4× pristine Co_3_O_4_; anti-humidity performance reported	[127]

Note: Response definitions are indicated in parentheses where available. S = R_a_/R_g_ for n-type, S = R_g_/R_a_ for p-type, or percentage change as defined in the original reference. Numerical values should not be compared directly across studies without considering the definition, concentration, and test conditions.

## 5. Approaches for Key Problems of Metal Oxide Sensors

### 5.1. Approaches to Reduce Operating Temperature of Metal Oxide Sensors

The high operating temperatures (typically 150–450 °C) required for conventional thermally activated metal oxide semiconductor gas sensors present significant challenges for coal mine deployment, including elevated power consumption, thermal management complexity, and intrinsic safety concerns in methane-containing atmospheres [41,55]. Recent research has focused on several promising strategies to achieve low-temperature or room-temperature operation while maintaining or enhancing sensing performance.

#### 5.1.1. Morphological Engineering and Nanostructuring

Reducing the dimensions of metal oxide sensing materials to the nanoscale provides a fundamental pathway for lowering operating temperatures. Nanostructured materials exhibit increased surface-to-volume ratios, enhanced surface reactivity, and reduced diffusion lengths for target gases, all of which contribute to improved low-temperature sensing performance [128,129].

Porous and Hierarchical Architectures: The creation of porous and hierarchical structures significantly enhances gas diffusion and provides abundant active sites for surface reactions at reduced temperatures. Porous In_2_O_3_ nanospheres developed by Xue et al. achieved effective methane sensing at 30 °C, maintaining normal response even under 90% relative humidity [72]. Similarly, hierarchical V_2_O_5_ nanoflowers synthesized by Mounasamy et al. demonstrated a distinct response of 8% toward 50 ppm methane at an exceptionally low operating temperature of 100 °C without requiring noble metal catalysts [86]. The enhanced performance was attributed to the high specific surface area, expanded internal voids, and abundant oriented surface active sites inherent to the hierarchical architecture.

One-Dimensional Nanostructures: One-dimensional architectures such as nanorods, nanowires, and nanobelts offer continuous electron transport pathways while maintaining high surface exposure. Belt-like In_2_O_3_ with mesoporous morphology, developed via electrospinning by Kgomo et al., achieved methane detection at 100 °C with rapid response and recovery times of 36 and 44 s, respectively [73]. The reduced operating temperature was attributed to the combined effects of morphological advantages and surface defect engineering.

#### 5.1.2. Noble Metal Catalytic Modification

Noble metal decoration represents one of the most effective strategies for reducing the operating temperature of metal oxide sensors through chemical and electronic sensitization mechanisms [58,59]. The catalytic spill-over effect of noble metals lowers the activation energy for target gas dissociation, enabling surface reactions at lower thermal energies.

Platinum and Palladium Modification: Haridas et al. demonstrated that Pd cluster-loaded SnO_2_ thin films achieved optimized methane sensing at a reduced operating temperature of 220 °C, significantly lower than conventional SnO_2_ sensors [76]. The enhanced performance was primarily attributed to the spill-over mechanism of Pd clusters, which lowered the activation energy for methane dissociation and accelerated surface reactions.

Silver and Gold Decoration: Liang et al. successfully fabricated Au-decorated vanadium oxide composite thin films achieving maximum methane response at room temperature, with sharp performance degradation at elevated temperatures due to metal-insulator transitions [88]. The enhanced low-temperature response was driven by high specific surface area, extensive oxygen vacancies, and a spill-over catalytic effect derived from Schottky contacts between the Au nanoparticles and the VOₓ matrix.

#### 5.1.3. Heterostructure Formation and Interface Engineering

The formation of heterojunctions between different metal oxides or between metal oxides and other functional materials creates built-in electric fields and modifies charge transfer kinetics, contributing to reduced activation barriers for gas-sensing reactions.

p-n Heterojunctions: Ni_2_O_3_-decorated SnO_2_ films developed by Vuong et al. demonstrated enhanced methane response at 400 °C, with the formation of effective nanoscale interfacial p-n heterojunctions at the p-Ni_2_O_3_/n-SnO_2_ boundary optimizing electron depletion dynamics [78]. Similarly, ZnO/NiO porous nanosheet composites synthesized by Zhang et al. exhibited remarkably enhanced gas-sensing capabilities at 340 °C compared to pure ZnO, achieving a 34.2% response toward 1000 ppm methane [81].

Organic–Inorganic Heterojunctions: Polyaniline/zinc oxide (PANI/ZnO) nanocomposites developed by Sen et al. demonstrated high response to methane at room temperature within a short response time of 20 s for 500 ppm methane [80]. This improvement was attributed to greater adsorption sites induced by large surface area and PANI/ZnO heterojunction effects with increased depletion depth due to gas molecule adsorption.

#### 5.1.4. Light-Assisted Sensing

Ultraviolet (UV) or visible light illumination provides an alternative energy source to thermal activation, enabling room-temperature operation while maintaining or improving sensing performance [54]. Light activation generates electron–hole pairs, promotes oxygen adsorption, and facilitates surface reactions without heating the sensor substrate.

UV-Activated Sensors: Sun et al. developed room-temperature ZnO methane sensors via UV illumination, where ZnO rods, plates, and spheres all clearly responded to methane under UV illumination but hardly responded in darkness [79]. The ZnO sphere device demonstrated the highest response and shortest response time, attributed to the hollow structure and more Zn atoms as active sites on the exposed (001) facet.

Mechanism of Photoactivation: Chizhov et al. systematically reviewed light-activated semiconductor gas sensors, concluding that photoexcitation can reduce operating temperatures by providing alternative reaction pathways through the generation of photogenerated charge carriers [54]. The photogenerated electrons and holes participate in surface reactions, lowering the energy barriers for oxygen adsorption and target gas oxidation.

#### 5.1.5. UV and Plasma-Assisted Synthesis

Beyond operational light activation, synthesis methods incorporating UV or plasma treatments can produce materials with enhanced low-temperature sensing properties through controlled defect engineering and surface modification [130].

UV-Assisted Synthesis: Controlled UV irradiation during material synthesis can generate oxygen vacancies and surface defects that serve as active sites for gas adsorption at reduced temperatures [131]. The UV-assisted synthesis approach has been shown to produce metal oxide materials with optimized defect concentrations and enhanced low-temperature reactivity.

Plasma Treatment: Plasma treatment of metal oxide surfaces introduces surface functional groups, modifies surface energy, and creates reactive sites that facilitate gas adsorption and reaction at lower temperatures [132,133]. This approach has been applied to various metal oxides to enhance their low-temperature gas-sensing properties.

### 5.2. Approaches to Enhance Selectivity of Metal Oxide Sensors in Complex Coal Mine Environments

Selectivity remains one of the most critical challenges for metal oxide semiconductor gas sensors in coal mine environments, where multiple interfering gases (H_2_, NH_3_, VOCs, H_2_S, SO_2_, NOₓ, CO_2_, and hydrocarbons) coexist with the target gases CH_4_ and CO [22,23]. Since both CH_4_ and CO are reducing gases that induce similar resistance decreases in n-type metal oxides, a single unmodified sensor cannot distinguish between them [37,52]. This section reviews the primary strategies developed to enhance selectivity, including catalytic filters, temperature modulation, array-based approaches, and machine-learning-assisted pattern recognition.

#### 5.2.1. Catalytic Filters and Chemical Selectivity Layers

Physical/Chemical Filters: The placement of selective catalytic filters or membranes in front of the sensing layer provides a passive approach to selectivity enhancement. These filters selectively remove or convert interfering gases before they reach the sensing surface while allowing target gases to pass through [134,135].

Selective Oxidation/Reduction Catalysts: Noble metal catalysts (Pt, Pd, Au, Ag) and transition metal oxides can be deposited as filter layers to promote preferential oxidation or reduction of specific interfering gases. For example, Pt-based catalysts have been shown to selectively oxidize H_2_ and CO while allowing CH_4_ to pass, based on differences in catalytic activity and operating temperature [136].

Zeolite and Molecular Sieve Filters: Zeolites and metal–organic frameworks (MOFs) with tailored pore sizes can act as molecular sieves, physically excluding larger interfering molecules while allowing smaller target gases to diffuse to the sensing surface [137]. The pore size and chemical functionality can be tuned to achieve selective gas permeation.

#### 5.2.2. Temperature Modulation and Pulsed Heating

Temperature-Programmed Sensing (TPS): By modulating the sensor operating temperature during measurement, different gases can be distinguished based on their distinct temperature-dependent response patterns [138]. Each gas exhibits characteristic response peaks at specific temperatures, enabling selective identification through temperature-resolved response profiles.

Pulse Heating Mode: Applying short heating pulses instead of continuous heating allows sensors to operate at different effective temperatures, with the response magnitude and kinetics providing information about the gas composition [139]. This approach has been successfully demonstrated for distinguishing between reducing gases in complex mixtures.

Thermal Desorption Analysis: Temperature-programmed desorption (TPD) techniques can be integrated with sensing measurements to analyze the desorption characteristics of different adsorbed gases, providing additional information for selectivity enhancement.

#### 5.2.3. Sensor Arrays and Electronic Nose Systems

Multi-Material Arrays: Arrays composed of multiple metal oxide sensing elements with different materials, dopants, or operating temperatures generate unique response patterns for different gases [140]. Ponzoni et al. demonstrated that an array of differently functionalized metal oxide sensors can effectively discriminate between various reducing gases based on their distinct response signatures [37].

N-Type and P-Type Complementary Sensors: Combining n-type and p-type metal oxide sensors provides complementary information because these materials exhibit opposite resistance changes upon exposure to reducing gases. The combination of n-type and p-type responses enhances the ability to distinguish between different gases [55,56].

MEMS-Based Multi-Sensor Platforms: Microelectromechanical system (MEMS) technology enables the integration of multiple sensing elements on a single chip, each with different materials, operating temperatures, or sensing modalities [83]. This approach is compatible with CMOS integration and facilitates the development of compact, low-power sensor arrays.

#### 5.2.4. Machine Learning and Pattern Recognition

Principal Component Analysis (PCA): PCA is widely employed to reduce the dimensionality of multi-sensor array data and visualize the distinct clusters corresponding to different gases [141]. This statistical approach enables qualitative and quantitative gas identification based on array response patterns.

Neural Networks and Deep Learning: Artificial neural networks (ANNs), support vector machines (SVMs), and deep learning algorithms have been increasingly applied to gas sensor array data for selective gas identification and concentration prediction [142]. These methods can learn complex patterns in sensor responses even in the presence of variable environmental conditions.

Feature Extraction: Critical features for machine learning classification include steady-state response magnitude, response/recovery kinetics, temperature-programmed response profiles, and frequency-domain characteristics from dynamic measurements [143]. Shooshtari and Salehi demonstrated that a carbon nanotube-titanium dioxide hybrid nanostructure-based electronic nose could effectively detect and discriminate volatile organic compounds using advanced pattern recognition techniques [68].

Real-Time Classification: Edge computing and lightweight machine learning models enable real-time gas classification directly on sensor nodes, reducing communication latency and power consumption [28]. Giri et al. demonstrated an IoT-enabled real-time warning framework for simultaneous CH_4_ and CO monitoring in coal mines [28].

#### 5.2.5. Multi-Modal Sensing Approaches

Combined Chemiresistive and Other Modalities: Integrating chemiresistive measurements with other sensing modalities (e.g., optical, electrochemical, thermal) provides additional information dimensions for gas discrimination [21]. Li et al. demonstrated a near-infrared wide-range dual-gas sensor system for simultaneous CH_4_ and CO detection in coal mine environments [21].

Dual Parameter Sensing: Simultaneously measuring multiple physical parameters (resistance, capacitance, impedance, and work function) from the same sensing material can provide complementary information for gas identification [144]. This approach exploits the different ways in which various gases affect these parameters.

Light- and Temperature-Modulated Sensing: Combining light activation with temperature modulation enables additional selectivity dimensions, as different gases exhibit distinct responses to both optical and thermal stimulation [54]. This multi-stimulus approach provides richer response patterns for pattern recognition.

#### 5.2.6. Selectivity Challenges for Specific Mine Interferents

Hydrogen (H_2_): H_2_ is a common interferent in coal mines, often generated during coal oxidation and battery charging operations. H_2_ exhibits high reactivity on metal oxide surfaces and can produce responses similar to CH_4_. Selectivity against H_2_ requires catalysts or filters that promote CH_4_-specific reactions or suppress H_2_ oxidation [145].

Ammonia (NH_3_): NH_3_ is generated during the decomposition of nitrogen-containing compounds in coal and can interfere with gas sensing. NH_3_ exhibits reducing properties similar to CH_4_ but displays distinct temperature-dependent response profiles and can be distinguished through temperature modulation [146].

Hydrogen Sulfide (H_2_S): H_2_S is a highly toxic and corrosive gas that can be generated during coal oxidation and microbial activity. H_2_S exhibits strong affinity for metal oxide surfaces and can poison sensing elements at high concentrations [147]. Selectivity against H_2_S requires surface engineering to minimize strong chemisorption.

Volatile Organic Compounds (VOCs): Coal mining operations release various VOCs, including alkanes, aromatics, and oxygenated compounds. VOCs generally exhibit slower response kinetics and higher desorption temperatures compared to CH_4_ and CO, enabling differentiation through temperature-programmed sensing [148].

Sulfur Dioxide (SO_2_) and Nitrogen Oxides (NOₓ): SO_2_ and NOₓ are generated during coal combustion and oxidation processes. These gases exhibit oxidizing properties and can induce resistance changes opposite to reducing gases, providing a basis for discrimination [149].

Carbon Dioxide (CO_2_): CO_2_, while not a significant interferent for most metal oxide sensors due to its low surface reactivity, can affect baseline resistance through physical adsorption and competitive effects on oxygen adsorption. Compensation or reference sensors are often required for accurate measurements in high-CO_2_ environments [150].

#### 5.2.7. Practical Implementation Considerations

Environmental Factors: Selectivity enhancement strategies must account for variable temperature, humidity, pressure, and ventilation conditions in underground coal mines [45]. Calibration models should incorporate environmental compensation to maintain selectivity under changing conditions.

Long-Term Stability: Selectivity strategies based on catalytic filters or surface modifications must maintain effectiveness over extended periods. Catalyst poisoning, filter degradation, and surface contamination can compromise selectivity over time [151].

Power and Integration Constraints: Selectivity enhancement strategies should be compatible with low-power, MEMS-based sensor platforms for practical deployment in coal mines. Temperature modulation and machine learning approaches require careful optimization to balance selectivity gains with power consumption.

### 5.3. Oxygen Compensation Strategies for Mine Deployment

Beyond material optimization and selectivity enhancement, practical deployment of MOS sensors in coal mines requires specific strategies to address oxygen-dependent performance variations. These strategies can be categorized into hardware-based and software-based approaches:

#### 5.3.1. Hardware-Based Compensation

Integrated Oxygen Sensors: Co-locating an electrochemical or optical oxygen sensor with the MOS gas sensor enables direct oxygen concentration measurement. The oxygen reading can then be used as an input for compensation algorithms. This approach requires additional sensor hardware but provides the most direct compensation path.

Dual-Material Reference Elements: Fabricating an additional MOS sensing element that is specifically optimized for oxygen response (rather than target gas detection) provides a reference signal that correlates with oxygen variations. This reference element can be located on the same chip using MEMS fabrication, minimizing temperature and environmental differences.

Gas Permeation Membranes: Selective gas permeation membranes can be applied to the sensor surface to control oxygen access or provide oxygen enrichment, reducing the effective oxygen dependence. However, these membranes must be carefully designed to avoid impeding target gas diffusion.

#### 5.3.2. Software-Based Compensation

Multi-Parameter Calibration Models: Mathematical models incorporating oxygen concentration, temperature, humidity, and pressure as independent variables can compensate for baseline and response variations. These models typically require extensive characterization data but can be implemented in embedded firmware.

Adaptive Baseline Tracking: Algorithms that track baseline resistance trends and adjust reference values dynamically can partially compensate for slow oxygen variations. However, these algorithms must be carefully designed to avoid tracking actual gas concentration changes.

Machine Learning Compensation: Neural networks or Gaussian process regression models trained on comprehensive environmental data can predict and compensate for oxygen-induced variations without explicit mechanistic models. These approaches can capture complex, non-linear interactions between multiple environmental factors.

#### 5.3.3. Operational Protocols

Periodic Auto-Calibration: Exposing sensors to reference gas mixtures (including defined oxygen levels) at regular intervals allows recalibration to compensate for drift induced by oxygen variations.

Ventilation Monitoring Correlation: Integrating sensor readings with mine ventilation monitoring data enables contextual interpretation, distinguishing between oxygen-induced baseline shifts and actual gas concentration changes.

### 5.4. How to Select a Sensor for Mine Use

The comparison indicates that there is no single “best” MOS material for every mine condition. For early CH_4_ warning, low-temperature or light-assisted materials are attractive because they reduce heater power and thermal risk, while porous/hierarchical and catalytic heterostructures can provide stronger responses at moderate temperatures. For CO, several materials achieve detection at or below the cited 50 ppm safety level, including room-temperature composites and low-temperature In_2_O_3_-, V_2_O_5_-, CuO-, and Co_3_O_4_-based systems. Nevertheless, the strongest laboratory response is not necessarily the best field choice. Selection should proceed in the following order: (1) verify the target concentration range and alarm margin; (2) verify CH_4_/CO and interferent discrimination; (3) evaluate humidity and oxygen dependence; (4) compare response/recovery and long-term drift; (5) assess power, temperature, and explosion-safety constraints; and (6) verify packaging, calibration, and communication requirements.

### 5.5. Future Perspectives: From Materials to Intelligent Mine-Safety Systems

Future development should move from isolated high-response materials toward integrated multi-gas-sensing systems. AI-assisted classification can combine partially selective MOS elements to distinguish CH_4_, CO, and interferents; edge computing can process sensor-array signals locally and reduce communication latency; wireless sensor networks can provide spatially distributed monitoring; digital-twin frameworks can combine gas concentration, ventilation, and mine-state information for risk prediction [152]. Self-powered, flexible, and wearable sensors may extend monitoring to distributed or difficult-to-access locations, although their durability and intrinsic safety must be demonstrated. MEMS/CMOS integration remains particularly important because it can reduce heater power, shrink device dimensions and enable multi-temperature or multi-material arrays. The most credible pathway is therefore a calibrated, low-power, multi-sensor platform rather than reliance on one highly sensitive sensing film.

## 6. Conclusions and Perspectives

This review shows that the most useful metal-oxide semiconductor sensor for coal-mine safety cannot be selected from sensitivity alone. CH_4_ and CO have different surface-reaction kinetics: CH_4_ is chemically stable and requires C–H bond activation, whereas CO is more readily oxidized on activated oxide surfaces. As a result, methane sensing generally benefits from catalytic activation, defect engineering, porous architectures, heterojunctions and low-temperature or light-assisted strategies, while CO sensing can often achieve strong responses at lower temperatures. Representative data in Table 3 and Table 4 demonstrate substantial differences in operating temperature, concentration range, response magnitude, and response/recovery time and also show that reported response metrics are not directly interchangeable.

For mine deployment, the central unresolved problem is selectivity under realistic mixed-gas and environmental conditions. Because CH_4_ and CO are both reducing gases, a single conventional MOS resistance signal is generally insufficient to identify them simultaneously. Multi-material arrays, catalytic filters, temperature/light modulation, complementary p-/n-type elements, and pattern-recognition or machine-learning methods therefore provide a more realistic route to selective multi-gas monitoring. Cross-sensitivity to H_2_, H_2_S, SO_2_, NO_x_, CO_2_, NH_3_, hydrocarbons, VOCs, and humidity should be evaluated at realistic concentration ratios, while oxygen dependence must be considered in poorly ventilated areas.

The mine environment also changes the definition of a “high-performance” sensor. A practical device must combine adequate margin below relevant alarm concentrations, rapid response, stable calibration, low drift, resistance to humidity/dust/condensation/vibration, low power consumption, and intrinsically safe packaging. The cited 2% CH_4_ and 50 ppm CO thresholds illustrate why ppm-level laboratory detection is useful but is not, by itself, evidence of mine readiness. Future work should therefore report standardized performance metrics under controlled variations of humidity, oxygen concentration, temperature, ventilation, and interferent gases.

Finally, the most promising development direction is the integration of optimized MOS materials into low-power MEMS/CMOS multi-sensor platforms. AI-assisted gas classification, edge computing, wireless sensor networks, digital-twin mine models, self-powered devices, and distributed multi-gas monitoring can convert material-level sensing advances into actionable mine-safety information. The principal contribution of this review is thus a reader-oriented selection framework: the preferred sensing approach is the one that achieves an acceptable balance of sensitivity, selectivity, response speed, power, stability, and environmental robustness under the actual constraints of underground coal mines.

## Figures and Tables

**Table 1:** Comparison among gas-sensing technologies for mines.

Technology	Strengths for Mine Monitoring	Main Limitations/Trade-Off
MOS chemiresistive	Low cost, compact, simple electrical readout, strong material tunability, compatible with arrays and MEMS/CMOS.	Cross-sensitivity, oxygen/humidity dependence, drift, and often elevated operating temperatures.
Electrochemical	Mature technology and useful selectivity for some gases; low-temperature operation.	Humidity/temperature sensitivity, finite electrolyte lifetime, and calibration/maintenance requirements.
Catalytic combustion/pellistor	Established methane-detection principle and direct relation to catalytic oxidation heat.	Catalyst poisoning/inhibition and heater-related power and safety considerations.
Optical/SPR	Fast optical response and potential high selectivity; can operate near room temperature.	More complex optical interrogation and packaging; not equivalent to a chemiresistive MOS device.
Photoacoustic	Strong spectroscopic selectivity and multi-gas capability.	Higher system complexity, optical/acoustic components, and power/cost considerations.

**Table 3:** Comparisons of representative CH*_4_* sensors discussed in this review.

Material/Architecture	CH_4_ Test Concentration	Operating T	Reported Response (and Definition)	LOD	Response/Recovery	Practical Observation	Reference
Porous In_2_O_3_ nanosheets	500 ppm	190 °C	S = R_a_/R_g_ (high, study-specific)	---	---	30-day stability; porous structure	[71]
Porous In_2_O_3_ nanospheres	CH_4_ (study range)	30 °C	S = R_a_/R_g_ (normal response)	---	---	Response retained at 90% RH	[72]
Belt-like In_2_O_3_	90 ppm	100 °C	S = R_a_/R_g_ = 1.1	---	36/44 s	Low-temperature operation	[73]
Ni-doped In_2_O_3_	200 ppm	140 °C	S = R_a_/R_g_ = 72.727	---	---	Strong response enhancement vs. pristine In_2_O_3_	[74]
Ag-doped In_2_O_3_	500 ppm	120 °C	S = R_a_/R_g_ = 27.5	---	---	Lower operating temperature; >100% improvement vs. pristine In_2_O_3_	[75]
WO_3_/SnO_2_ nanoflowers	500 ppm	110 °C	S = R_a_/R_g_ (2.3× pristine SnO_2_)	38 ppb	---	Low LOD and heterojunction enhancement	[77]
ZnO sphere + UV	CH_4_ (reported concentrations)	Room T + UV	S = R_a_/R_g_ (highest among tested ZnO morphologies)	---	---	Light-assisted sensing avoids continuous high-temperature heating	[79]
PANI/ZnO	500 ppm	Room T	S = R_a_/R_g_ (high response)	---	20 s response	Room-temperature hybrid sensor	[80]
V_2_O_5_ nanoflowers	50 ppm	100 °C	S (%) = 8% (R_a_/R_g_)	---	---	Low-temperature CH_4_ activation without noble metal	[86]
V_2_O_5_ nanorods	4000 ppm	50 °C	S (%) = 23% (R_a_/R_g_)	---	Rapid	Low-temperature operation; reported selectivity	[87]
PANI/Co_3_O_4_ core–shell	500 ppm	Room T	S (%) = 8.73% (R_g_/R_a_)	---	---	Room-temperature operation	[97]
NiO nanoflakes	30 ppm	225 °C	S = R_g_/R_a_ = 46.53	---	15/20 s	Linear response reported from 0.2 to 50 ppm	[91]

Note: Response definitions are indicated in parentheses where available. *S* = *R_a_/R_g_* for n-type, *S* = *R_g/R_a* for p-type, or percentage change as defined in the original reference. Numerical values should not be compared directly across studies without considering the definition, concentration, and test conditions.

## Data Availability

No new data were created or analyzed in this study. Data sharing is not applicable to this article.

## Abbreviations

**Abbreviation**	**Full Form**
AC	Activated Carbon
AFM	Atomic Force Microscopy
ANN	Artificial Neural Network
BET	Brunauer–Emmett–Teller
CFD	Computational Fluid Dynamics
CMOS	Complementary Metal-Oxide Semiconductor
CNC	Coral-like Nanochain
CO	Carbon Monoxide
CVD	Chemical Vapor Deposition
DFT	Density Functional Theory
DOS	Density of States
DRIFTS	Diffuse Reflectance Infrared Fourier Transform Spectroscopy
EDL	Electron Depletion Layer
EDS	Energy-Dispersive X-ray Spectroscopy
FESEM	Field Emission Scanning Electron Microscopy
GIXRD	Grazing Incidence X-ray Diffraction
HAL	Hole Accumulation Layer
HOMO-LUMO	Highest Occupied Molecular Orbital–Lowest Unoccupied Molecular Orbital
HRTEM	High-Resolution Transmission Electron Microscopy
IoT	Internet of Things
LOD	Limit of Detection
MEMS	Microelectromechanical System
MO	Metal Oxide
MOF	Metal–Organic Framework
MOS	Metal Oxide Semiconductor
PANI	Polyaniline
PCA	Principal Component Analysis
PLAL	Pulsed Laser Ablation in Liquid
PNR	Porous Nanorod
rf	Radio Frequency
RH	Relative Humidity
SCS	Solution Combustion Synthesis
SEM	Scanning Electron Microscopy
SMT	Semiconductor-to-Metal Transition
SPR	Surface Plasmon Resonance
SVM	Support Vector Machine
TEM	Transmission Electron Microscopy
TPD	Temperature-Programmed Desorption
TPS	Temperature-Programmed Sensing
TVO	Titanium-Vanadium Oxide
UV	Ultraviolet
VOC	Volatile Organic Compound
XPS	X-ray Photoelectron Spectroscopy
XRD	X-ray Diffraction
